# Differential Activation of Diverse Glutathione Transferases of *Clonorchis sinensis* in Response to the Host Bile and Oxidative Stressors

**DOI:** 10.1371/journal.pntd.0002211

**Published:** 2013-05-16

**Authors:** Young-An Bae, Do-Whan Ahn, Eung-Goo Lee, Seon-Hee Kim, Guo-Bin Cai, Insug Kang, Woon-Mok Sohn, Yoon Kong

**Affiliations:** 1 Department of Molecular Parasitology, Sungkyunkwan University School of Medicine and Center for Molecular Medicine, Samsung Biomedical Research Institute, Suwon, Korea; 2 Department of Microbiology, Graduate School of Medicine, Gachon University, Incheon, Korea; 3 Department of Molecular Biology and Biochemistry, School of Medicine, Kyung Hee University, Seoul, Korea; 4 Department of Parasitology and Institute of Health Sciences, Gyeongsang National University College of Medicine, Jinju, Korea; Khon Kaen University, Thailand

## Abstract

**Background:**

*Clonorchis sinensis* causes chronic cumulative infections in the human hepatobiliary tract and is intimately associated with cholangiocarcinoma. Approximately 35 million people are infected and 600 million people are at risk of infections worldwide. *C. sinensis* excretory-secretory products (ESP) constitute the first-line effector system affecting the host-parasite interrelationship by interacting with bile fluids and ductal epithelium. However, the secretory behavior of *C. sinensis* in an environment close to natural host conditions is unclear. *C. sinensis* differs from *Fasciola hepatica* in migration to, and maturation in, the hepatic bile duct, implying that protein profile of the ESP of these two trematodes might be different from each other.

**Methodology/Principal Findings:**

We conducted systemic approaches to analyze the *C. sinensis* ESP proteome and the biological reactivity of *C. sinensis* glutathione transferases (GSTs), such as global expression patterns and induction profiles under oxidative stress and host bile. When we observed *ex host* excretion behavior of *C. sinensis* in the presence of 10% host bile, the global proteome pattern was not significantly altered, but the amount of secretory proteins was increased by approximately 3.5-fold. Bioactive molecules secreted by *C. sinensis* revealed universal/unique features in relation to its intraluminal hydrophobic residing niche. A total of 38 protein spots identified abundantly included enzymes involved in glucose metabolism (11 spots, 28.9%) and diverse-classes of glutathione transferases (GSTs; 10 spots, 26.3%). Cathepsin L/F (four spots, 10.5%) and transporter molecules (three spots, 7.9%) were also recognized. The universal secretory proteins found in other parasites, such as several enzymes involved in glucose metabolism and oxygen transporters, were commonly detected. *C. sinensis* secreted less cysteine proteases and fatty acid binding proteins compared to other tissue-invading or intravascular trematodes. Interestingly, secretion of a 28 kDa σ-class GST (Cs28σGST3) was significantly affected by the host bile, involving reduced secretion of the 28 kDa species and augmented secretion of Cs28σGST3-related high-molecular-weight 85 kDa protein. Oxidative stressors induced upregulated secretion of 28 kDa Cs28σGST3, but not an 85 kDa species. A secretory 26 kDa μ-class GST (Cs26μGST2) was increased upon treatment with oxidative stressors and bile juice, while another 28 kDa σ-class GST (Cs28σGST1) showed negligible responses.

**Conclusions/Significance:**

Our results represent the first analysis of the genuine nature of the *C. sinensis* ESP proteome in the presence of host bile mimicking the natural host environments. The behavioral patterns of migration and maturation of *C. sinensis* in the bile ducts might contribute to the secretion of copious amounts of diverse GSTs, but a smaller quantity and fewer kinds of cysteine proteases. The Cs28σGST1 and its paralog(s) detoxify endogenous oxidative molecules, while Cs28σGST3 and Cs26μGST2 conjugate xenobiotics/hydrophobic substances in the extracellular environments, which imply that diverse *C. sinensis* GSTs might have evolved for each of the multiple specialized functions.

## Author Summary

*Clonorchis sinensis* is a trematode parasite that infects the hepatobiliary ducts of the mammals including humans. Approximately 35 million people are infected and 600 million people are at risk of infections. Epidemiological studies have convincingly demonstrated relationships between clonorchiasis and cholangiocarcinoma. *C. sinensis* is a Group 1 biological carcinogen. *C. sinensis* excretory-secretory products (ESP) constitute the first-line effector system affecting the host-parasite interrelationship. We observed global expression pattern of *C. sinensis* ESP in the presence of host bile/oxidative stresses, which mimics natural host environments. The secretory proteome displayed common and unique features, which might be related to its habitat in the definitive host. The universal proteins were found to be several enzymes involved in glucose metabolism, glutathione transferases (GSTs) and oxygen transporters. *C. sinensis* differentially regulated the secretion of diverse GSTs in response to bile and oxidative stressors, which suggested that these enzymes are importantly involved in the protection from immune cell-derived oxidizing molecules and detoxification of hydrophobic substances. *C. sinensis* secreted less cysteine proteases, which play roles in tissue migration and immune evasion, compared to other tissue-invasive or intravascular trematodes. Our data suggest strongly that different GSTs might have differentially evolved with their specialized functions to cope with stressful conditions and ensure parasite's long-standing survival in the hosts.

## Introduction

*Clonorchis sinensis* is a trematode parasite that resides in the hepatobiliary tract of mammals including humans. Its enzootic infection is highly prevalent in several Asian countries including China, Vietnam, Japan and Korea. Approximately 35 million people are infected and 600 million people are at risk of infections worldwide [1], [2]. Humans are infected with *C. sinensis* by eating raw or undercooked cyprinoid fish harboring the infective metacercariae. When humans are lightly infected with *C. sinensis*, most are asymptomatic. However, chronic cumulative infections cause several symptoms associated with biliary ductal systems such as mechanical obstruction and stone formation. Histopathological alterations include ductal dilatation and periductal fibrosis combined with an adenomatous metaplasia. The most serious complication of clonorchiasis is associated with cholangiocarcinoma (CCA) [1], [3].

CCA results from malignant transformation of cholangiocytes. CCA is the second most common type of primary liver cancer [4], [5]. The etiology of CCA remains largely undetermined, but evidence indicates that the primary sclerosing cholangitis, parasitic infections and hepatitis are predisposing factors [6]. Epidemiological studies have convincingly demonstrated relationships between clonorchiasis and CCA; *Clonorchis*-infected individuals might die earlier than non-infected individuals due to the high incidence of CCA in areas where clonorchiasis is prevalent [7], [8]. *C. sinensis* has been classified as a Group 1 biological carcinogenic agent [9].

*C. sinensis* thrives for over 10 years in humans. To ensure its long survival within the bile ducts, where hydrophobic substances, immune effector molecules and several kinds of glycosylated host enzymes are profuse [10], the fluke continuously releases bioactive molecules to cope with the cytopathic environments. The proteinaceous and non-proteinaceous components secreted by the parasite, referred as excretory-secretory products (ESP), are intimately involved in biological processes including the induction of immune compromise and evasion, parasite feeding and detoxification/neutralization of host-derived cytotoxic molecules. Effects of helminth ESP might be reciprocal. ESP might promote proliferation of epithelial cells through its stimulant effects, which accompanies changes in gene expression patterns [11]–[13]. Conversely, ESP might contribute to the creation of an anti-inflammatory micromilieu, where hostile host effector cells prevail. Antioxidant enzymes, molecules involved in local immune regulation and cysteine proteases likely play critical roles during these interactions [14]–[16].

Glutathione transferases (GSTs) are a group of highly versatile and multifunctional enzymes that principally function in conjugation of glutathione to electrophilic donors, thereby participating in the detoxification processes, especially in helminth parasites. Several different types of acidic/neutral cytosolic-, μ-, σ- and ω-class GSTs have been characterized in helminth parasites [17]. Most helminth GSTs are expressed in the parasite's parenchyme and subteguments, which suggests either that they interact with physiologically/pathologically active molecules during the phase II detoxification process, or that these enzymes are secreted and directly contact the host's defensive system [18]. Crystal structure and molecular modeling studies have revealed critical differences in their substrate and/or inhibitor specificity according to xenobiotic substrate-binding sites [19], [20], which implies that these isozymes might mediate different cellular homeostatic processes.

A total of four GST isozymes have been recognized in *C. sinensis*. A 28 kDa σ-type CsGST (Cs28GST; AF051318) is expressed in the subtegument and underlying mesenchymal tissues [21]. Two cDNAs that encoding cytosolic 28 kDa GSTs (CsGST, DQ179264 and CsGST1, DQ342327), whose substrate specificity and inhibitor profiles of the recombinant enzymes were partly analyzed, have been isolated, but their exact identities remain elusive [22], [23]. A 26 kDa GST (cs26GSTM; L47992), having a folding topology similar to the μ-class GSTs, can conjugate GSH to the reactive carbonyl of peroxidized lipids [24]. (Protein names of each GST are adapted from original authors). However, no systemic approaches have yet been undertaken to analyze the biological reactivity of CsGSTs, such as global expression patterns and induction profiles under oxidative stress and host bile. Moreover, the secretory behavior of *C. sinensis* in an environment close to natural host conditions has remained with uncertainty.

In the present study, we comparatively analyzed the *ex host C. sinensis* ESP proteome obtained in the presence and absence of the rabbit bile. ESP obtained in the presence of the host bile might represent the authentic nature of the *C. sinensis* ESP, because *C. sinensis* resides in the bile-filled hepatobiliary ducts of a definitive host. *C. sinensis* differs from *Fasciola hepatica* in migration to, and maturation in, the hepatic bile duct, implying that the proteome array of *C. sinensis* ESP might be different from that of *F. hepatica*. We observed that incubation medium supplemented with host bile substantially augmented the parasite's secretion, but retained its compositional profile. Interestingly, release of σ- and μ-classes of GSTs was differentially regulated in response to the host bile and oxidative stressors.

We provide the basis for identification of genuine *C. sinensis* excretory-secretory proteome, which might be critically involved in the pathobiological alterations, thus significantly deepening our understanding of this clinically important human pathogen.

## Materials and Methods

### Ethics Statement

All animals were housed in accordance with guidelines from the Association for the Assessment and Accreditation of Laboratory Animal Care (AAALAC). All protocols for animal infections were approved by the Institutional Review Board and conducted in the Laboratory Animal Research Center of Sungkyunkwan University (protocol 2008-8-18).

### Parasite and Rabbit Bile

*C. sinensis* metacercariae were collected from naturally infected freshwater fish, *Peudorasbora parva*, in an endemic area in Jinju, Gyeongsangnam-do, Korea. Three New Zealand White rabbits were orally infected with 500 metacercariae/rabbit using a gavage needle. The adult worms were recovered from rabbit bile ducts at 8-week post-infection. The worms were washed more than 10 times with physiological saline at 4°C. Bile fluid was aseptically drawn from gall bladders of three age-matched rabbits and centrifuged at 20000 g for 30 min at 4°C to remove host-derived cell debris.

### Preparation of *C. sinensis* Samples

Viable intact worms were collected under a dissecting microscope. The worms were placed in serum-free RPMI-1640 medium (pH 7.2; Life Technology) at 37°C for 1 h to ensure emptying of the guts. The flukes were incubated in serum-free RPMI-1640 medium in the absence or in the presence of rabbit bile (1%, 5%, 10% or 20%) or hydrogen peroxide (0.5 mM) at 37°C for 1 h (20 worms/group/3 ml medium). Incubations of dead but intact worms, which were prepared by treating the worms for 10 min at 55°C in RPMI-1640 medium with/without 10% host bile, were included as negative controls. After removing the worms, the conditioned medium was centrifuged at 3000 g for 5 min followed by 20000 g for 30 min at 4°C. Resulting supernatants were used as ESP. After incubation, the worms were washed three times with ice-chilled PBS (100 mM, pH 7.2) and were immediately used in the extraction of proteins and RNAs. All procedures were repeated in triplicate. ESP were also prepared in a large scale (10 ml) with 100 worms, to obtain sufficient samples for the identification of secreted proteins as well as to minimize variation caused by different physiological conditions of individual worms. Protein profiles in an equal volume and/or an equal amount of protein contents of the experimental ESP samples were examined by 12% SDS-PAGE under reducing conditions and/or by 2-dimensional electrophoresis (2-DE) as described below.

### Identification of Secretory Proteome of *C. sinensis* ESP

ESP were precipitated with ice-chilled 10% tricholoacetic acid/acetone and resuspended with rehydration buffer containing 6 M urea, 2 M thiourea, 2% CHAPS, 0.4% DTT, 0.5% IPG buffer (pH 3–10; GE Healthcare) and 0.002% bromophenol blue (BPB). The proteins were separated along with the respective isoelectic points on IPG strips with a cup-loading instrument (Amersham Biosciences) for a total of 35 kVh, followed by 12% SDS-PAGE (160×160×1 mm). Protein spots were stained with colloidal Coomassie Brilliant Blue G-250 (CBB). The spots of interests were excised from the 2-DE gels and were processed with in-gel tryptic digestion. The tryptic peptide mixtures were analyzed with an AutoflexII matrix-assisted laser desorption/ionization-time of flight mass spectrometry (MALDI-TOF MS) system (Bruker Daltonics, GmbH). The peptide mass fingerprints (PMFs) and tandem MS obtained from each spot were searched against the NCBI protein sequence database (http://www.ncbi.nlm.nih.gov) and locally mounted *C. sinensis* EST and draft genome database [25], [26] using Biotools software (Bruker Daltonics). The PMF tolerance was ±100 ppm and tandem MS tolerance was ±0.8 Da. Single missed cleavage site, cysteine carbamidomethylations and methionine oxidation were considered. Protein identifications were based on Mascot scores that were above the significant threshold, which was set at *p*<0.05. Protein probability was assigned by the Protein Prophet algorithm [27]. For the MALDI-TOF/TOF MS analysis, all of the spectra were obtained using a long-lifetime N2 laser, operating in nitrogen flow-through mode (20 Hz frequency), yielding laser irradiation at λ = 337 nm with a maximal laser power of approximately 110 µJ. Typically 500–800 shots of MS acquisition were summed for the acquisition of a laser-induced dissociation spectrum at elevated laser power. Bruker Biotools software was used to interpret and annotate the tandem MS. Database searches were conducted with the Matrix Science Mascot search engine program.

### Determination of Chromosomal Structures of *CsGSTs*

*C. sinensis* genomic DNA was isolated using a Wizard Genomic DNA Purification Kit (Promega). The chromosomal segments of *Cs28σGSTs* (AF051318 and DQ342327) were amplified from the genomic DNA employing long-range PCR. The primers were designed from the nucleotide sequences within both ends of each cDNA (CsGSTs-g-F and CsGSTs-g-R; Table S1). The amplification reactions were done with the LA *Taq* system under a thermal cycling profile recommended by the manufacturer (Takara) and analyzed on an agarose gel. The amplicons cloned into pGEM-T Easy vectors (Promega) were sequenced and compared with their corresponding cDNA sequences to determine the positions and lengths of intervening introns. The general rules for splice site consensus sequences were considered to verify the accuracy of exon-intron boundary regions (gt-ag). *O. viverrini* DNA was isolated from the lyophilized worms, which were a generous gift from Dr. B Insisiengmay (Department of Hygiene and Prevention, Ministry of Public Health, Lao PDR).The genomic structure of *O. viverrini* ortholog (AY057838) was similarly determined using the genomic DNA and a primer pair (Ov28GST-g-F/R; Table S1).

### Generation of Recombinant GST Proteins and Specific Mouse Antisera

The open reading frame (ORF) regions of *Cs26μGST* and *Cs28σGST* genes amplified by PCR from an adult *C. sinensis* cDNA library with gene-specific primers (Table S1) were cloned into the pET-28a expression vector and transformed into competent *Escherichia coli* BL21 (DE3). The expression of recombinant proteins was induced by adding 0.5 mM isopropyl-1-thio-β-D-galactopyranoside (IPTG) into the bacterial cell cultures. The recombinant proteins were purified by nickel-nitrilotriacetic acid (Ni-NTA) affinity chromatography (Qiagen) and monitored by 12% reducing SDS-PAGE. Polyclonal antibodies were generated by immunizing BALB/*c* mice with respective recombinant GST proteins mixed with Freund's adjuvants (Sigma-Aldrich) according to the standard protocol. Final booster was done by intravenous injection of 10 µg proteins. The blood was collected by heart puncture, centrifuged for 10 min at 3000 g and the respective antisera were stored at −80°C until use.

### *In vitro* Induction Pattern of CsGSTs in Response to Different Exogenous Stimuli

Fresh intact worms were pre-incubated in 50 ml serum-free RPMI-1640 medium for 1 h at 37°C. The worms (20 worms/group/3 ml medium) were transferred into the fresh medium containing methyl viologen dichloride hydrate (Paraquat; 25 and 100 mM), 5-hydroxy-1,4-naphthoquinone (Juglone; 25 and 100 µM) or hydrogen peroxide (H_2_O_2_; 0.5 and 2 mM), followed by 1 h incubation at 37°C. The soluble proteins from the respective conditioned worms were separately prepared by homogenizing the worms in PBS (100 mM, pH 7.2) followed by centrifugation at 20000 g for 30 min at 4°C. The conditioned medium and proteins extracted from worms incubated without oxidative treatment were taken as controls. The induction profiles of CsGSTs were examined through Western blots probed with each of the CsGST-specific mouse antisera. An anti-cathepsin F antibody available in our laboratory was used as a quantity/quality control, of which constant expression was verified by reverse transcription (RT)-PCR, in association with the respective stimuli (unpublished observation). Intact viable adults were also incubated with rabbit bile (1–20%) for 1 h at 37°C and the induction profiles of *CsGSTs* were assessed with the worm extracts and ESP. Three independent experiments were done with freshly prepared worms.

### Western Blot Analysis

Proteins separated by SDS-PAGE/2-DE were transferred to nitrocellulose membranes (Schleicher & Schuell, BioScience). The membranes were blocked in Tris-buffered saline (100 mM, pH 7.6) supplemented with 1% Tween 20 (TBST) and 5% skim milk, after which membranes were incubated with the respective anti-CsGST antibodies (1∶1000 dilution of each) and subsequently with horseradish peroxidase-conjugated goat anti-mouse IgG antibody (1∶4000 dilution). The signals were detected using an ECL detection system (Amersham Biosciences). For quantitative analysis, all images were exposed for 1 min.

### Real-Time Quantitative Reverse Transcription-PCR (real-time qRT-PCR)

Total RNAs were extracted from adult *C. sinensis* with the TRIzol reagent (Invitrogen). First-strand cDNA was synthesized by reverse transcribing 3 µg of total RNAs in a final reaction volume of 100 µl using a RNA PCR Kit (AMV, ver2.1; Takara) and an oligo(dT) 15 primer. The cDNAs were used in the following PCR procedure to amplify each of the antioxidant genes in 96-well plates. The PCR mixtures contained 5 µl of diluted cDNAs (1∶5), 5 µl of 2× SYBR Green PCR Master Mix (Applied Biosystems) and 200 nM of each gene-specific primer (Table S1). Real-time qRT-PCRs was performed in a thermocycling profile (95°C for 5 min followed by 40 cycles of 15 s at 95°C and 1 min at 60°C) employing ABI Prism 7000 Sequence Detection System and software (PE Applied Biosystems). Control reactions were done with RNAs that had not been reverse transcribed to verify absence of genomic DNAs in the RNA samples. All of the reactions were independently conducted in triplicate. The specificity of the amplicons was verified by melting curve analysis at a temperature ranging from 60–95°C, after observing the PCR products by agarose gel electrophoresis. The data were normalized against the values obtained with the *β-actin* gene (EU109284) and the fold-induction in the experimental groups was calculated by comparison of those of the non-stimulated controls.

## Results

### Changes of Global Excretion-Secretion Behavior of *C. sinensis* by Exogenous Host Bile, Oxidative Chemical or Incubation Time

We incubated fresh intact adult worms in RPMI-1640 at 37°C for 1 h in the presence or absence of host bile. The conditioned media were examined by 12% SDS-PAGE to observe the effects of host bile on *C. sinensis* excretory-secretory behavior. *C. sinensis* released numerous proteins that were mainly 18–30 kDa and 50–90 kDa. The banding profiles were highly reproducible in three different batches according to increasing doses of host bile (a representative data set is shown in Figure S1A). However, the relative intensity of each band and the total amounts of the proteins were substantially increased in the bile-induced ESP in a dose-dependent manner, in which approximately 3.5- and 5-fold increase were observed in the 10%- and 20%-bile ESP, respectively (Figure S1B). Addition of 0.5 mM H_2_O_2_ into the incubation medium also augmented the protein secretion, especially of the 20–30 kDa species. Time lapse analysis indicated that incubation of *C. sinensis* up to 8 h in RPMI-1640 did not induce considerable alteration in the protein profile, but the total amount of ESP and the relative intensity of the respective bands were substantially increased in proportion to the incubation time (data not shown), as previously observed with *Fasciola hepatica* [13], [28].

**Figure 1:**
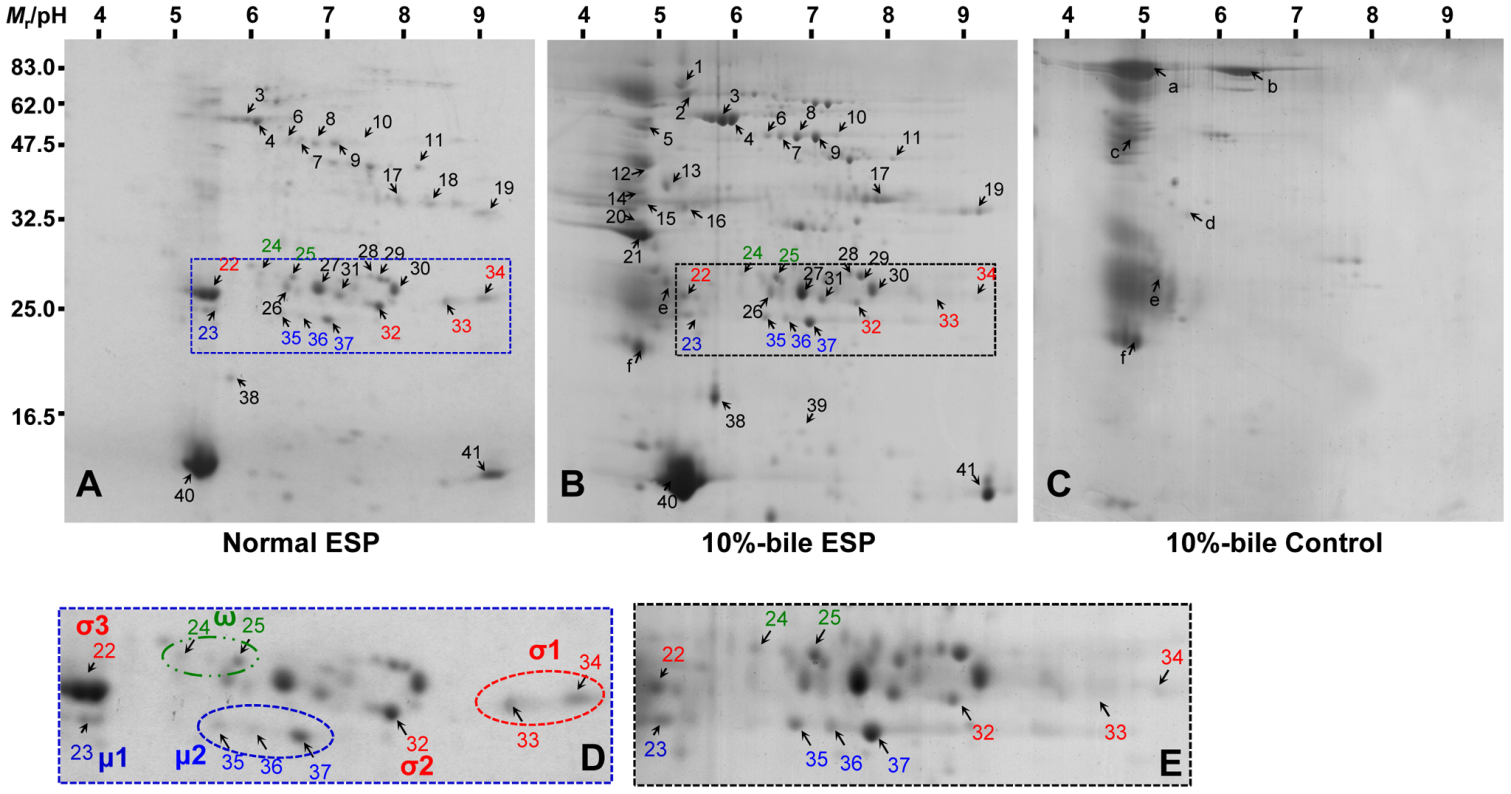
Two-dimensional proteomic array of *C. sinensis* ESP. One hundred worms were incubated in RPMI-1640 (normal ESP) or in RPMI-1640 containing 10% rabbit bile (10%-bile ESP) at 37°C for 1 h. ESP precipitated with 10% tricholoacetic acid/acetone were resuspended with rehydration buffer. (**A**) The normal ESP (200 µg proteins) was isoelectrofocused (pH 3–10) and further separated by 12% SDS-PAGE. (**B**) The 2-DE gel image obtained with loading of the equal protein amounts of the 10%-bile ESP. (**C**) The 2-DE analysis of the same volume of culture medium of dead worm in the presence of 10% rabbit bile. (**D**) and (**E**) Protein spots with significantly intensified or diminished in the presence of bile are indicated by boxes and are highlighted. Protein spots were visualized by CBB staining and further processed with MALDI-TOF MS or TOF-TOF identification. The spots identified are marked by an arrow and Arabic numeral (see also Table 1 and Table S1). Proteins identified as GST isoforms are marked by red (σ-class), blue (μ-class) and green (ω-class) letters.

We detected more than 100 protein spots on CBB-stained 2-DE gel of normal ESP (ESP obtained by incubating the worms in the absence of host bile), in which 200 µg proteins were loaded (Figure 1A). These spots were evenly distributed across a broad pH range of 5–10 and the relative intensities were similar to one another, except for spots 22, 27, 40 and 41. The profile appeared to be relatively well maintained in the same concentrations of 10%-bile ESP, although the spots in the acidic pH region became complicated by superimposition of bile proteins (Figures 1D and 1E). Total amounts of proteins in normal and bile-induced ESPs showed a certain degree of variation in the three separate preparations (<20%). Nevertheless, overall spotting patterns were comparable among the preparations (data not shown).

These collective data suggested that global excretion-secretion pattern of *C. sinensis* was not significantly affected by the elapsed time either after being removed from the host or after being exposed to the *in vitro* stresses. Rabbit bile, which comprises the natural host environment of *C. sinensis*, was cytopathic against the parasite, even when used in concentrations of 1–20% (v/v), probably because the bile juice collected from normal rabbit gall bladder were enriched with secretory IgA and mucin of up to 10-fold concentration [29]. We maintained *in vitro* incubations for 1 h in the presence of 10% bile juice to minimize the adaptive changes and degeneration of the worms during incubation in *ex host* conditions.

### *C. sinensis* ESP Proteome Reveals Three Major Fractions Composed of Enzymes Involved in Glucose Metabolism, GSTs and Cysteine Proteases

The protein identities were determined by MALDI TOF/TOF MS or MALDI TOF-MS, and following Mascot analyses against the non-redundant proteomic database of GenBank and locally mounted EST and draft genome database [25], [26]. A total of 193 protein spots (138 from 10%-bile ESP and 55 from normal ESP) together with 15 from 10%-bile control were selected for the identification. Most spots showed a good PMF score, but many spots could not be precisely identified during the searches. Moreover, although a great deal of information exists in genomic and proteomic databases for *S. japonicum* and *F. hepatica*, these databases did not match well with protein spots detected in *C. sinensis* ESP. We were able to identify 38 protein spots from the normal and 10%-bile ESP, of which three protein spots (5, 12 and 15) were originated from rabbit and six proteins from 10%-bile control (spots a–f, Figure 1C). The *C. sinensis* proteins were largely categorized into three major groups: Glycolytic enzymes (11 spots, 28.9%), antioxidant GSTs (10 spots, 26.3%) and proteolytic enzymes (four spots, 10.5%) (Table 1. See also Table S2 for detailed description).

**Table 1:** Protein identification of adult *C. sinensis* ESP and 10% bile juice.

Spot no(s).	MS^a^	Theoretical *M*_r_/pI	Description: Protein function^b^	Sig. pept.^c^
**Proteins identified from 10%-bile ESP**				
1, 2	TT	73058/5.13	Paramyosin: Myofibril and myosin filament	−
3, 4	MT	20670/6.93	Aldehyde dehydrogenase: Oxidoreductase	−
5	TT	83835/4.92	Polymeric Ig-receptor: Unknown	+
6, 8–10	TT	46245/6.28	Enolase: Glycolysis	−
11	MT	75090/5.22	Hypothetical protein: Unknown	−
12, 15	TT	83835/4.92	Polymeric Ig-receptor: Unknown	+
14	TT	44118/7.74	Myosin heavy chain: Musculoskeletal/ATP-binding	−
16	TT	36520/5.20	Cathepsin F: Proteolysis	+
17	MT	36355/8.16	GAPDH^d^: Glycolysis	−
18	MT	51537/5.40	Succinate-semialdehyde dehydrogenase: Glycolysis	−
19	MT	36432/8.57	Malate dehydrogenase: Carbohydrate metabolism	−
21	MT	21813/5.76	LOH1CR12^e^: Unknown	−
22	MT	24735/5.22	**Cs28σGST3** f: Oxidative stress	−
23	MT	25470/6.74	**Cs26μGST1**: Oxidative stress	−
24, 25	MT	34814/6.49	**Cs28ωGST**: Oxidative stress	−
26	MT	48399/4.78	SJCHGC05999: Ca^++^-dependent proteolysis	−
27, 30, 31	MT	25756/4.97	Cathepsin F: Proteolysis	+
28	MT	27064/8.18	Triosephosphate isomerae: Glycolysis	−
29	MT	27064/8.18	Triosephosphate isomerae: Glycolysis	−
32	MT	24559/6.97	**Cs28σGST2**: Oxidative stress	−
33, 34	MT	24775/8.40	**Cs28σGST1**: Oxidative stress	−
35–37	MT	25080/6.07	**Cs26μGST2**: Oxidative stress	−
38	MT	19596/5.51	Ferritin: Iron homeostasis	−
39	TT	10949/9.85	SJCHGC01414: DNA-binding	−
40	MT	16985/5.14	Myoglobin: Oxygen transporter	−
41	MT	20490/9.45	Fatty acid binding protein: Lipid binding/transporter	−
**Proteins identified from 10%-bile ESP**				
a, c	TT	84975/4.92	Polymeric Ig-receptor: Unknown	+
b	MT	70861/5.85	Serum albumin precursor: Albuminoid superfamily	+
d	MT	36213/5.42	Annexin A4: Calcium-binding	−
e	MT	24097/7.64	Junction adhesion molecule 2: Cellular adhesion	+
f	MT	16003/4.72	Immunoglobulin J chain: Polymeric Ig-polypeptide	−

a Mass spectra were observed by MALDI-TOF/TOF (TT) or MALDI-TOF (MT).

b Protein functions based on Gene Ontology of UniProtKB (http://uniprot.org).

c Presence of signal peptide sequence predicted by the SignalP 4.0 (http://www.cbs.dtu.dk/services/SignalP/) and PSORT (http://psort.nibb.ac.jp).

d Glyceraldehyde 3-phosphate dehydrogenase.

e Loss of heterozygosity, 12, chromosomal region 1 protein homolog.

f *C. sinensis* GSTs renamed in this study by considering their identification order, biochemical properties, phylogenetic positions and locations on 2-DE gel are indicated by bold characters.

The primary fractions of the proteins contained enzymes involved in the glycolysis such as enolase (spots 6 and 8–10), glyceraldehyde-3-phosphate dehydrogenase (spot 17) and triosephosphate isomerase (spots 28 and 29), or in Krebs cycle (malate dehydrogenase; spot 19). Spot 7 was related with *F. hepatica* enolase, but the Mascot value of 37 was not significant (significant value is >43). Mitochondria-type aldehyde dehydrogenases (spots 3 and 4), which are responsible for the conversion of toxic aldehydes into non-toxic compounds, and succinate-semialdehyde dehydrogenase (spot 18), which participates in the degradation of γ-aminobutyric acid, were additionally identified.

A total of 10 GST family members including the μ-, σ- and ω-classes were detected in the molecular weights (*M*_r_) and isoelectric point (pI) ranges of 26–28 kDa and 5.2–9.3, respectively (Figures 1D and 1E). The PMF of the 28 kDa molecules matched to those of previously identified *C. sinensis* GSTs; spot 22 (pI 5.2) to CsGST1 (ABC72085), spot 32 (pI 7.5) to CsGST (ABA56496), and spots 33 and 34 (pIs 8.4 and 9.0) to Cs28GST (AAD17488) (Table 1 and Table S2). Protein spot 22 (pI 5.2) was identical to parenchymal-type CsGST1 (ABC72085) [23]. This protein was not confined intracellularly, but was secreted. Interestingly, its expression was significantly decreased in the presence of 10% host bile. Spot 32 (pI 7.5) was identified as another σ-class GST (CsGST; ABA56496) [22]. Spots 33 and 34 (pI 8.4 and 9.0) were closely related to Cs28GST (AAD17488) [21]. We renamed these σ-type GSTs as Cs28σGST3 (acidic protein), Cs28σGST2 (neutral protein) and Cs28σGST1 (basic proteins), respectively, in accordance with their discovery order. The multiple 26 kDa proteins with pI 6.5 (spots 35–37) were identical to μ-type cs26GSTM1 (AAB46369) [24]. Spot 23 (pI 5.2) displayed a PMF that matched to *Echinococcus multilocularis* μ-type GST (CAA59739). We designated spots 35–37 as Cs26μGST2 and spot 23 as Cs26μGST1. The PMF analysis further identified spots 24 and 25 orthologous to the *Schistosoma mansoni* ω-type GST (AF484940) [30], which was newly recognized in *C. sinensis* ESP proteomes (Figure 1D and Table 1).

The cathepsin L-like cysteine proteases were a common type of proteolytic enzyme released by *C. sinensis* (spots 16, 27, 30 and 31). Their *M*_r_ (26–28 kDa) indicated these proteases were secreted after proteolytic maturation; however, spot 16, which was detected only in bile ESP, had a large *M*_r_ (>32 kDa) and exhibited PMF matched to the ‘Inhibitor I29’ (^42^TYSNDDDELRFEIFK^56^) conserved in the prodomain of cathepsin F [31].

Each of two spots homologous to paramyosin, myosin and aldehyde dehydrogenase, as well as one spot each representing ferritin, myoglobin and fatty acid-binding protein were detected. Protein spots of 13 and 20 were associated with *S. mansoni* myosin II heavy chain (CAA46548; *M*_r_/pI of 61585/5.20) and *S. japonicum* metalloprotease (AAX27148; *M*_r_/pI of 32683/8.43), while they showed only one peptide hit. In addition, a portion of the tryptic peptides obtained from some other protein spots revealed good PMF score or MS spectra, but homologous identities could not be retrieved from the GenBank databases due mainly to their low sequence coverage. To further validate the applicability of our *in vitro* incubation system, we determined the protein profile of 10%-bile control. Multiple rabbit proteins released into bile were detected, with the identities of poly-immunoglobulin-receptor, albumin, annexin A, adhesion molecules and immunoglobulin J-chain (Figure 1C and Table 1). These proteins, except for spots e and f, were hardly detected in the bile ESP, probably due to the proteolytic degradation induced by *Clonorchis* proteases and/or to the superimposition of numerous proteins within the narrow acidic region (Figure 1B).

### *C. sinensis* Secreted Diverse Classes of GSTs, among which σ-class GSTs Have Undergone Duplication Events to Expand Paralogous Gene Pool

Analysis of primary and tertiary structure of Cs28σGSTs revealed that amino acid residues involved in the GSH binding and enzymatic catalyst were highly conserved in the *Clonorchis* protein and their homologs (Figure S2). Cs26GSTs exhibited distinct patterns of amino acid conservation with several μ-type GSTs (data not shown) similar to those of schistosome orthologs [32]. Nevertheless, the recombinant forms of Cs26μGST2, Cs28σGST1 and Cs28σGST3 demonstrated comparable biochemical properties in regard to pH and temperature optima and inhibitor sensitivity (Figure S3). The close relationship between trematode μ- and σ-class GSTs was further evaluated by a cladistic analysis of 239 GST members retrieved from the protostomian databases of the GenBank, using the neighbor-joining algorithm of MEGA. The phylogenetic tree separated these proteins into distinct clades, consistent with the types classified by the original authors (Figure S4). The major portion of the protostomian GSTs deposited in the databank seemed to belong to the σ-class, which were allocated well into separate clusters according to the taxonomies of their donor organisms at the class level. The platyhelminth GSTs positioned in the clades proximal (σ-, α- and π-like proteins) or identical (μ-like proteins) to the μ- and π-class members of ecdysozoans, although some of the branching nodes were not statistically significant. This unique phylogenetic relationship would partly support the overlapping enzymatic properties of several trematode GSTs.

**Figure 2:**
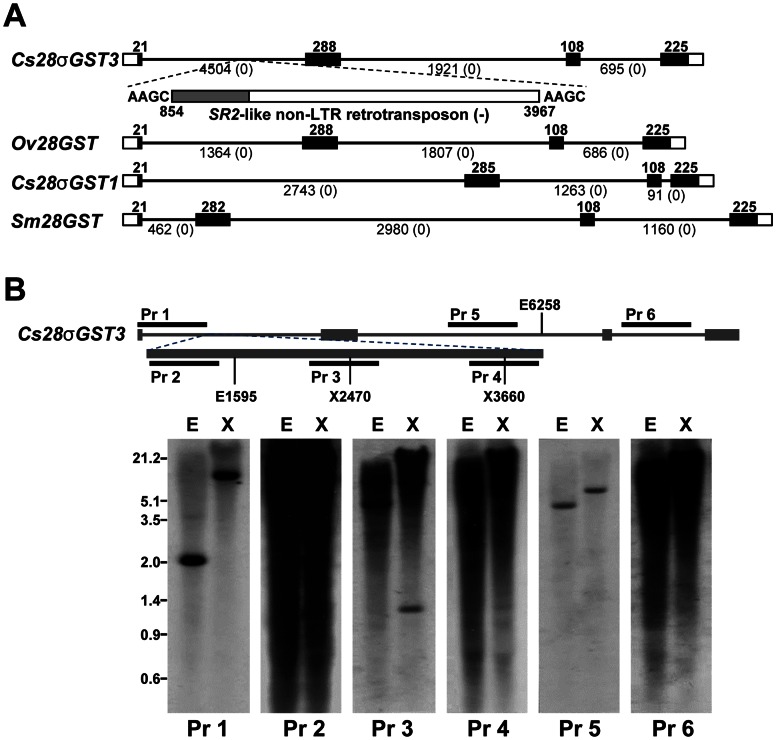
Genomic structure and distribution pattern of *Cs28σGST3* and *Cs28σGST1*. (**A**) The chromosomal structures of *Cs28σGST3* and *1* are compared to their trematode orthologs retrieved from *Opisthorchis viverrini* (*Ov28GST*) and *Schistosoma mansoni* (*Sm28GST*) genomes. The open and solid squares indicate the untranslated and ORF regions, respectively, and the intervening intorns are represented with solid lines. The Arabic numerals demonstrate the size of each exon and intron. The intron phase is presented in parenthesis. A non-long terminal repeat retrotransposon inserted in the first intron of *Cs28σGST3* is marked with a square (the gray-colored region in the square corresponds to an ORF of the element for reverse transcriptase). The nucleotide positions and duplicated target site are also marked at both ends of the element. (**B**) The genomic DNAs of *C. sinensis* restricted with *Eco*RI (E) and *Xho*I (X) were resolved in a 0.8% agarose gel and transferred onto nylon membranes. The membranes were hybridized with dioxygenin-labeled probes, which were prepared from various regions of *Cs28σGST3*, as indicated at the top. The positive signals were developed with a Chemiluminescent Dig Detection System.

The phylogenetic analysis suggested that the genic dosage of σ-type GST has been preserved differently among trematode genomes. Analysis of *S. mansoni* and *S. japonicum* genomic databases in the Sanger Institute (http://www.sanger.ac.uk/) and Shanghai Center for Life Science & Biotechnology Information (http://lifecenter.sgst.cn/) indicated the single-copy dosage of schistosome orthologs (data not shown), while the copy number could not be properly estimated in *C. sinensis*. The evolutionary relationships among the trematode σ-class GST genes were further examined by comparing their genomic structures, which were isolated from respective genomes either by PCR amplification (*C. sinensis* and *O. viverrini*) or *in silico* screening (*S. mansoni*). These genes exhibited an identical structural topology with four exons and three introns. Their respective intervening introns occupied orthologous positions, despite the significant length polymorphism (Figure 2A). BLAST searches detected a *SjR2*-like non-long terminal repeat (LTR) retrotransposon [33] sporadically integrated in the first intron of *Cs28σGST3*, which contributed to the length polymorphism of *Cs28σGST3*. Southern blot analysis of *C. sinensis* genomic DNAs with multiple probes specific to various regions of *Cs28σGST3* demonstrated that the other introns were also evolved *via* insertional events of repetitive genetic elements of as-yet-unidentified repetitive elements (Figure 2B). The chromosomal segment of *Sm26μGST* was composed of seven exons and six introns, but none was orthologous to those of the σ-type GST genes (data not shown). These collective data suggest that either *C. sinensis* σ-class GST lineage has undergone duplication events to expand this paralogous gene pool in *C. sinensis*, unlike schistosome genomes.

### Expression Pattern of *C. sinensis* GST Proteins

**Figure 3:**
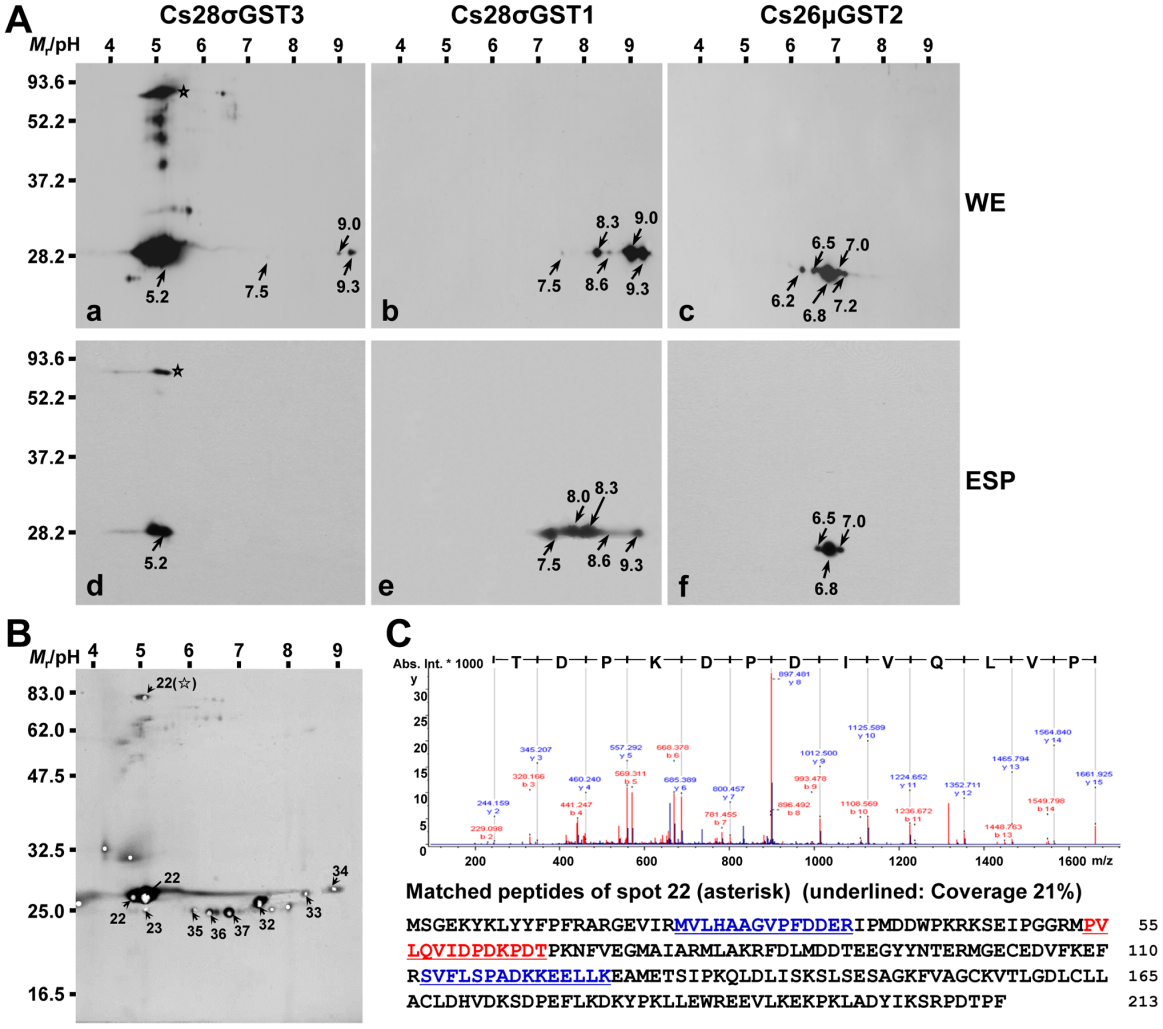
Detection and purification of GSTs expressed in adult *Clonorchis sinensis*. (**A**) Worm extracts (50 µg protein) and normal ESP (20 µg protein) were resolved by 2-DE (pH 3–10) and 12% SDS-PAGE. The proteins were electroblotted to nitrocellulose membranes and incubated with polyclonal mouse antibodies specific to the respective recombinant GSTs (1∶1000 dilutions) and subsequently with HRP-conjugated anti-mouse IgG antibody (1∶4000 dilutions). The positive signals were developed with an ECL detection kit. In case of Cs28σGST3, 10 µg ESP proteins were used to detect the signal. The pI values of major signals were marked in the Western blotting images. (**B**) Global purification of GST proteins from the worm extracts employing GSH-affinity column. Spot numbers are same as shown in Figure 1. (**C**) Tandem mass spectrum of the peptide fragments of spot 22 (m/z = 897.481) shown in **B**. The specific fragment sequence is matched with ^54^PVLQVIDPDKPDT^66^ of the Cs28σGST3. In addition, ^22^R.MVLHAAGVPFDDER.I^35^ (Oxidation of M, ions score 70) and ^113^R.SVFLSPADKKEELLK.E^127^ (ions score 58) were also recognized by MALDI-TOF searches.

A previous study demonstrated that Cs28GSTs might play a major role in antioxidant activity compared to Cs26GSTs because molar ratio of these two proteins approximated 14∶1 in adult *C. sinensis* [21]. The protein array revealed that σ-class GSTs constituted the major GST fractions secreted by *C. sinensis* in the absence of bile juice (Figure 1D). The conditions were parallel with global GST expression profile of *C. sinensis* parenchyme, which was purified using a GSH-affinity column and following 2-DE analysis (Figure 3B). Conversely, 10%-bile ESP showed a critical difference, in which the release of Cs28σGST3 (spot 22) was significantly decreased, while those of Cs26μGST2 (spots 35–37) were increased (Figure 1E). These results suggested strongly that the functional expression and secretion of GSTs might be different in response to exogenous stimuli including bile. To delineate the expression pattern of these GSTs, we cloned Cs28σGST1 and 3 (AAD17488 and ABC72085) and Cs26μGST2 (AAB46369), which exhibited significantly upregulated or downregulated secretion in the presence or absence of host bile. We expressed these GSTs in *E. coli* cells and generated monospecific antibodies that did not cross react with one another. We examined the expression pattern of these GSTs in the normal ESP (20 µg) and the worm extracts (50 µg) employing these antibodies. As shown in Figure 3A, when worm extracts were probed with anti-recombinant Cs28σGST3 antibodies, protein spots of ca. 56 and 85 kDa were detected in addition to a prominent 28 kDa spot (The reactions at 41 and 47 kDa were not consistent in other experiments). The 28 kDa protein was abundantly expressed in the parenchyme and, in part, released into the ESP (Figure 3A, panels a and d). Cs28σGSTs with pIs 9.0 and 9.3 were the major fractions in the parenchyme, while those with pIs 8.0 and 8.3 constituted main ESP fractions. Considering the identification profiles (Table 1) and the tightly conserved regions within their primary structures (Figure S2A), spots seen above pH 9.0 would be Cs28σGST1-related proteins and those with pH 8.0 were likely to be homologous to Cs28σGST2 (Figure 3A, panels b and e). A small amount of isoforms generated *via* posttranslational modification, or unidentified paralogs might be secreted, as seen by their different reaction intensities at different pIs on Western blotting. Cs26μGST2 fractions were located at neutral pH (6.5–7.0) and most of these proteins appeared to be secreted (Figure 3A, panels c and f).

Due to highly complicated pattern including superimposition of proteins of the bile ESP, we were unable to identify a GST-related high-molecular-weight-protein at 85 kDa (Figure 1B), which were clearly observed in immunoblotting (Figures 3A, panels a and d, denoted by asterisks). The global CsGST fractions were purified through a GSH-affinity column from the worm extracts. The 26 and 28 kDa proteins separated into multiple spots according to their respective pI values (Figure 3B), which showed similar patterns to those of excretory GSTs in the absence of bile. These spots were indeed identified as the same molecules identified in the ESP (spot numbers are same as in Figure 1). We detected an 85 kDa protein with an acidic pI (Figure 3B, asterisk). The protein was identified as Cs28σGST3 by MALDI-TOF/TOF MS analysis (Figure 3C). The protein also demonstrated a high PMF score with the *O. viverrini* 28 kDa GST (Ov28GST; AL2371). Spot(s) corresponding to Cs28σGST2 could not be detected in the fractionated GSTs, probably owing to the relatively low expression level of the protein (Figure 3A, panel b). The protein spots corresponding to the secretory ω-class GSTs (spots 24 and 25; Figures 1D and 1E) were not purified by GSH-affinity chromatography (Figure 3B), which was relevant to the minimal affinity of the class members toward agarose-coupled GSH [30].

### Oxidative Stress-Induced Secretions of CsGST Proteins

**Figure 4:**
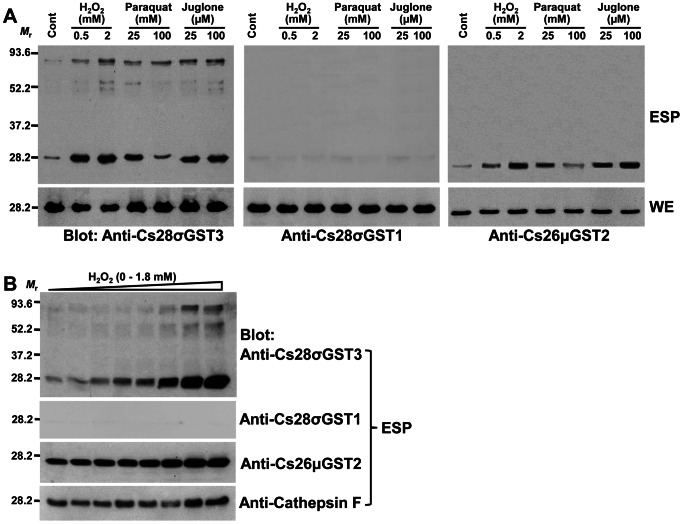
Induction profiles of *C. sinensis* GST proteins under oxidative stresses. (**A**) The fresh viable flukes were transiently confronted with oxidative stresses by incubating in 1 ml RPMI-1640 supplemented with hydrogen peroxide (0.5 and 2 mM), paraquat (25 and 100 mM) and juglone (25 and 100 µM) for 1 h at 37°C. ESP (20 µl) and worm extracts (WE; 30 µg) were resolved by 12% reducing SDS-PAGE, blotted to nitrocellulose membranes and probed with specific antibodies against the respective recombinant proteins of Cs26μGST2, Cs28σGST1 and Cs28σGST3. Positive signals were detected with an ECL detection kit. (**B**) The worms were stimulated with hydrogen peroxide (0–1.8 mM) for 1 h at 37°C. The secretion of Cs28σGST3 and Cs26μGST2 in an equal volume of each ESP (20 µl) were subjected to Western blotting. Secretory cathepsin F was used as an internal control, which did not show any expressional changes upon oxidative stimuli.

The protein array of *C. sinensis* ESP indicated that diverse GSTs are released into the surrounding environments, which was strongly suggestive of their primary defensive roles in protecting the worms from exogenous toxic substances. The secretory CsGSTs were substantially increased in bile-induced ESP. However, the rabbit bile seemed to suppress the release of Cs28σGST3, which obviously contrasted to its upregulatory effects of secretion of other GST molecules (Figures 1D and 1E). We assessed the induction profile of the CsGSTs upon stimulation with oxidative chemicals. Oxidative stress conditions created by H_2_O_2_ and juglone significantly increased the release of Cs28σGST3 and Cs26μGST2 in a dose-dependent manner. Paraquat, a superoxide free radical generator, augmented secretion of Cs28σGST3 and Cs26μGST2 at low concentrations (25 mM), but inhibited their secretions at high concentrations (100 mM). However, secretory Cs28σGST1 was not induced in response to these oxidative stressors (Figure 4A, upper panels). The expression pattern of these three GST molecules was relatively constant in intracellular compartments (Figure 4A, lower panels). The decreased expression of Cs28σGST3 and Cs26μGST2 in the worms incubated with 100 mM paraquat might result from the lethal effects of the chemical at the high concentrations (approximately 60% of the worms survived after the treatment). We subsequently examined the excretion of GSTs upon treatment with increasing doses of H_2_O_2_. The secretion of Cs28σGST3 and Cs26μGST2 were increased significantly in a dose-dependent fashion, while that of Cs28σGST1 was hardly observed (Figure 4B). Secretory cathepsin F, which was used as a negative control, showed no expressional changes upon the oxidative stimuli. These collective data demonstrated that different GST species exerted their differential roles in response to different exogenous stimulus.

### Bile Augmented Secretion of High Molecular Mass Cs28σGST3, But Not Expression of *Cs28σGST3* Transcript

**Figure 5:**
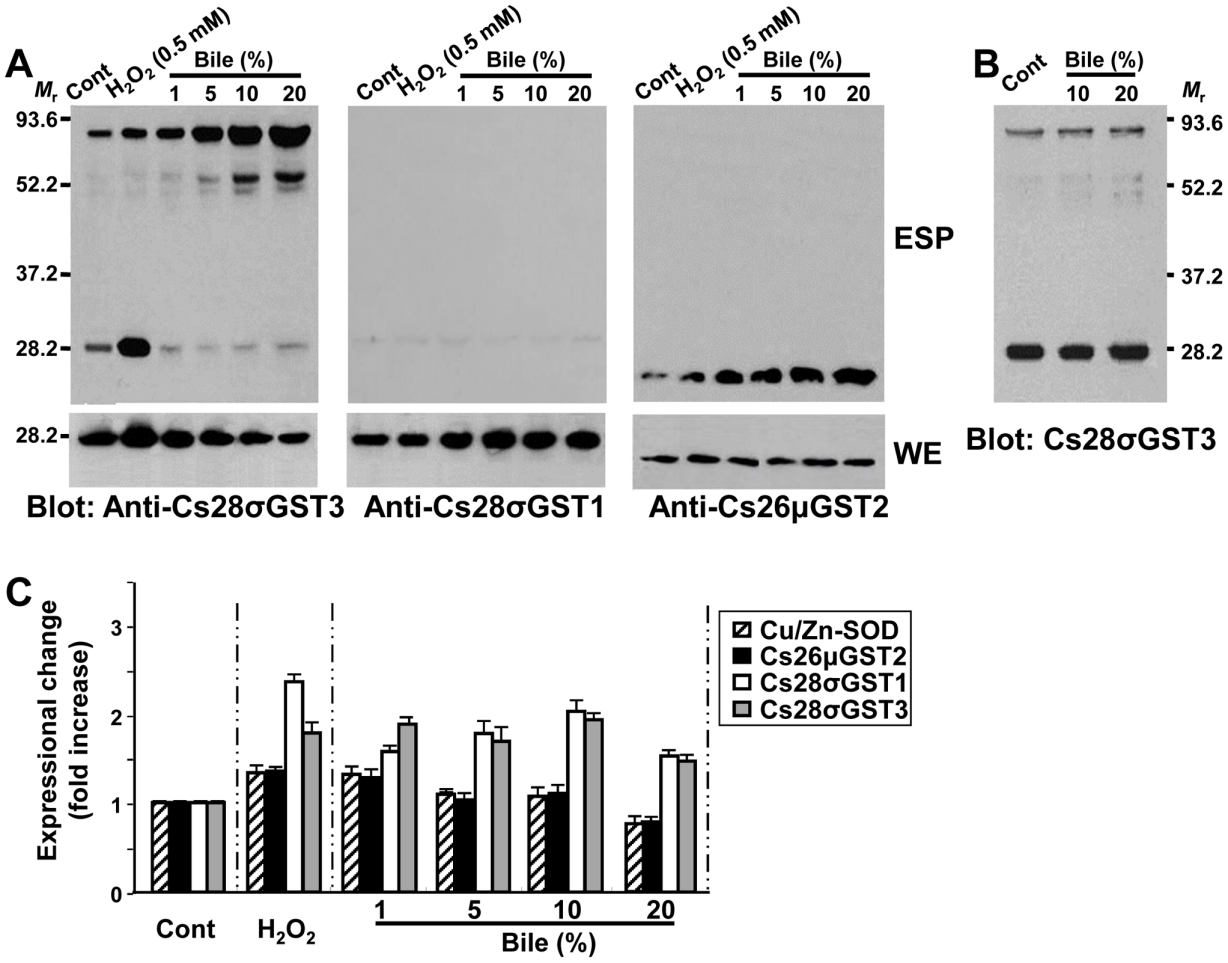
Effects of rabbit bile on the secretion and expression of *C. sinensis* GSTs. (**A**) Fresh viable adults were challenged with rabbit bile at various concentrations, as indicated at the top, for 1 h at 37°C (10 worms/group/1 ml medium). Incubations in RPMI-1640 only and 0.5 mM hydrogen peroxide-RPMI were included as a negative and positive control, respectively. The ESPs (20 µl) and worm extracts (WE; 30 µg) were analyzed by Western blotting employing each of the specific antibodies. Signals were detected by ECL reagents. (**B**) ESP obtained by incubating worm with RPMI-1640 only was incubated with RPMI or rabbit bile (10% and 20%) at 37°C for 1 h. The mixture was separated by 12% reducing SDS-PAGE and electroblotted. The membrane was probed with specific antibodies against the recombinant Cs28σGST3. The reaction was detected with ECL detection kit. (**C**) Changes of *CsGST* transcripts observed by quantitative real-time reverse transcription PCR. The worms obtained from a parallel experiment were harvested and subjected to total RNA preparation. The mRNA transcripts in each of the RNA samples were reverse-transcribed and the resulting cDNAs were employed in real-time PCR as templates to amplify anti-oxidant genes. A primer pair for β-actin gene was included in the reaction to normalize signals obtained by each of the selected genes. Induction profile of Cu/Zn-superoxide dismutase (SOD) was also included. The fold increase was calculated by comparing intensities between experimental and control (RPMI-1640 only) groups.

To scrutinize the expression and secretion pattern of CsGSTs upon bile stimulation, worm extracts obtained from cultures with rabbit bile were analyzed by Western blotting. As bile concentrations increased, the secretion of 85 kDa Cs28σGST3 species dose-dependently increased, whereas that of 28 kDa Cs28σGST3 decreased (Figure 5A, left panel). In addition, the 28 kDa Cs28σGST3 in the parenchyme appeared to be proportionally decreased along with increased secretion of 85 kDa Cs28σGST3 species. The expression of Cs28σGST1 was slightly augmented in the parenchyme, while secretion of Cs28σGST1 was negligible (Figure 5A, middle panel). Secretion of Cs26μGST2 was also increased to some extent, while its expression was not significantly modified in the intracellular compartment (Figure 5A, right panel). We then assessed whether the substantial increase of the secreted 85 kDa Cs28σGST3 was due to an abiotic cross-linking of the Cs28σGST3 monomer under the high concentrations of bile. We coincubated *C. sinensis* normal ESP with 10% and 20% rabbit bile for 1 h at 37°C, but found no significant polymerization effect (Figure 5B). We further analyzed whether this phenomenon was associated with the altered expression of these transcripts by real-time qRT-PCR. As shown in Figure 5C, incubation with the viable worms either in 0.5 mM H_2_O_2_ or in 1–20% rabbit bile did not significantly revise the expression of these transcripts, although those of Cs28σGST1 and Cs28σGST3 marginally increased and that of the Cs26μGST2 was slightly decreased as bile concentration increased. These results indicated the relative stability of the expression levels of these GSTs, but that *C. sinensis* might adjust its secretion behavior to cope with stressful conditions and ensure its survival in the hosts.

## Discussion

In the present study, we incubated fresh viable adult *C. sinensis* in the presence or absence of host bile in a relatively short period of 1 h to minimize possible alteration of secretory behavior through physiological adaptation during longer term incubation, as well as to mimic the natural host microenvironment. The overall protein expression pattern of *C. sinensis* was not significantly affected by the host bile, in terms of the spectrum of secretory molecules, but the total amount of secreted protein was substantially increased in the presence of 10% bile up to approximately 3.5-fold. This result suggests that bile components might continuously arouse *C. sinensis* and thus, promote secretion of ESP in the natural host environments. The ESP proteome primarily constituted with several enzymes, whose functions might be related mainly with energy metabolism and nutrition, detoxification, immune evasion and worm migration. Previous studies with *C. sinensis* and other trematode parasites such as *O. viverrini*, *F. hepatica*, *S. mansoni*, *S. japonicum* and *Paragonimus westermani* also demonstrated these proteins are plentiful in the ESP. These collective observations suggest that the adaptive biological processes and associated molecular networks might be tightly conserved in the parasitic trematodes to allow host-parasite interplay [13]–[16], [28].

Proteome analysis of the 10%-bile ESP demonstrated that some of the major GST proteins such as μ-, σ- and ω-classes were released into the surrounding environment. Secretions of these antioxidant proteins were largely inducible by stimulating worms with host bile or oxidants. However, as is shown in Figures 1D and 1E, secretion of 28 kDa Cs28σGST3 species appeared to be substantially decreased (up to 95%) in the bile stimulated ESP, while that of immunologically-related high-molecular weight proteins were profoundly augmented as bile concentrations increased. This is a unique finding observed for the first time with *C. sinensis* ESP, but not with other trematode such as *F. hepatica* and *O. viverrini*, which also reside in the mammalian bile duct [13], [16], [34]. We purified the global CsGST fractions using GSH-matrix and observed that the 85 kDa protein shared peptide fragments with the 28 kDa Cs28σGST3, thus representing a genuine Cs28σGST3. The high-molecular-weight-protein did not originate from a simple cross-linking process occurring abiotically between the 28 kDa protein and bile components (Figure 5B). Screening of the *C. sinensis* cDNA library with primers designed from nucleotide sequences of the conserved peptide fragments could not detect any genes with a larger size. Southern blotting of *C. sinensis* genomic DNA with the *Cs28sGST3*-specifc probes demonstrated that the parasite genome contained only a single copy gene homologous to *Cs28σGST3* cDNA (Figure 2B). Comprehensive analyses on the developmental changes of *C. sinensis* transcriptome also could not detect putative gene(s) encoding the 85 kDa protein homologous to the 28 kDa Cs28σGST3 [35], [36]. These collective data indicate that the 85 kDa protein represents the multimeric form of the 28 kDa Cs28σGST3 generated by an as-yet-unknown biochemical linkage. A recent study showed that oxidative stress modifies the multimerization of mouse homer scaffolding proteins through disulfide cross-linking [37]. A portion of many of the proteins form oligomeric complexes *via* inter-molecular disulfide bonds, which can easily be broken by treating the proteins with reducing agents such as DTT and β-mercaptoethanol. However, 85 kDa Cs28σGST3 appeared to be highly tolerant against these reducing agents. Mammalian bile contains enormous hydrophobic substances, such as cholesterol, phospholipids, fatty acids and triglycerides, in addition to proteins and hormones [10], [29], most of which influence *C. sinensis* survival. Therefore, it is conceivable that the parasite might sequester these toxic compounds by conjugating them on the secretory Cs28σGST3. Further studies are warranted to elucidate the physicochemical cross-linking among 28 kDa molecules or the plausible conjugation mechanism between Cs28σGST3 and bile component(s).

*S. japonicum* ESP abundantly contains a series of fatty acid binding proteins (SjFABPs) and heat shock proteins (HSP90α and HSP70) [15] while those of *F. hepatica* and *P. westermani* principally possessed diverse isoforms of cysteine proteases, among which cathepsin L/F are the major constituents. These molecules exert their main effects on lipid trafficking and assimilation, parasite feeding, migration and immune evasion in the hosts [13], [14], [28], [38]. Protein array of *C. sinensis* ESP revealed critical differences compared to those of *S. japonicum*, *F. hepatica* and *P. westermani*, which included secretion of diverse GSTs and relatively low-level secretion of proteases, HSPs and FABPs (Table 1). However, secretion of several GST isozymes was highly similar to *F. hepatica*. The different secretory behavior observed in these parasites might reflect the migration manners and favorable localities in their hosts. *C. sinensis* metacercaria excysts in the duodenum and directly migrates into the bile ducts through the ampulla of Vater within a few days of infection [39] and thrives therein, with its entire lifespan being over 10 years. *C. sinensis* does not penetrate any host tissues/organs and are hardly exposed to host immune system, which implies that the parasite might secrete effector molecules to attenuate cytopathic bile components. Therefore, the biological significance of the GSTs of *C. sinensis* and *F. hepatica*, whose functions are mainly associated with protection from immune cell-derived oxidizing molecules, as well as conjugation, sequestration and detoxification of hydrophobic substance, would be substantially critical to ensure their survival. Secretion of μ- and σ-class GSTs was indeed significantly and specifically accelerated in juvenile *F. hepatica* that migrated through the liver, compared with the pre- and post-liver stage [40]. On the other hand, *F. hepatica* and *P. westermani* travel to their main habitat through the penetration of several internal organs/tissues of the host, during which they continuously interact with host immune effector system [13], [38], [40]. The unique migration route of *C. sinensis* might contribute to secretion of a small quantity and fewer types of cysteine proteases, whose main function is related to tissue hydrolysis and immune evasion. ESP proteome of *O. viverrini*, which has similar migration behavior and residing niche in the definitive hosts, also revealed significant proportions of antioxidant GSTs but very low amounts of cysteine proteases [16].

*C. sinensis* secretes copious amounts of myoglobin, which functions as an oxygen reservoir and an intracellular oxygen carrier. Helminth parasites perform anaerobic respiration in the host environments [41]. Probably, *C. sinensis* expresses a large amount of myoglobin to guarantee maximal uptake of oxygen in the extremely anaerobic environmental niche. Alternatively, the protein may carry out an essential role in scavenging of nitrosative stress molecules generated by host inflammatory cells infiltrating the ductal epithelium [42]. An excretory-secretory myoglobin of *Trichostrongylus colubriformis* was shown to induce protective immunity against challenging infections following a single intraperitoneal injection [43]. The biological reactivity of *C. sinensis* myoglobin needs further researches.

Long-standing inflammations in the bile ducts, which are associated with helminthic infections such as *C. sinensis* and *O. viverrini*, induced hyperplasia and malignant transformation of the cholangiocytes [5], [9], [44], [45]. The inflammatory and oxidative conditions provoked by the parasite infections might trigger genomic instability in hepatobiliary ductal cells, which can lead to the neoplastic transformation *via* destructive damages on the chromosomal DNAs and proteins [16], [46]. Cytokines, free radicals and growth factors released by infiltrated host immune cells are also involved in generation of the inflammatory and tumorous conditions [47], [48]. Finally, restorative hyperplasia in tissues damaged by both direct contact of the parasite to the bile duct epithelium and irritation by the bioactive ESP molecules further promotes the propagation of pre-malignant cells, which are more vulnerable to a carcinogen [45]. Only small proportions of the helminthic infections such as *O. viverrini* and *C. sinensis* are related with CCA tumorigenesis. Liver fluke infections remain a major public health problem, especially in high endemic areas, given the large number of people who are exposed to and/or infected [2], [44], [49] and the strongest link between CAA compared to other bacterial/viral infections [5], [8], [9]. More attention should be paid on the prevention and control of this carcinogenic parasite.

In order to investigate secretory proteins concerning their physiological functions, a full range of biochemical, immunological and proteomic analyses should be conducted employing ESP collected in the genuine host environments. However, the natural ESP is not readily collectable from most of parasites, particularly due to the high turnover rate of secreted proteins or their extremely low concentrations [13], [15]. We incubated *C. sinensis* in RPMI-1640 medium supplemented with 10% bile for a relatively short period (1 h) to minimize the effects of tegumental turnover and worm degradation [50]. However, we detected multiple proteins, which are believed to be targeted into cytosol such as cytoskeletal proteins (paramyosin and myosin heavy chain) and glycolytic enzymes (enolase, glyceraldehyde-3-phosphate dehydrogenase and triosephosphate isomerase). We also detected prodomain of the cathepsin F. Furthermore, a large proportion of proteins identified in the *C. sinensis* ESP did not contain the classical signal peptides (Table 1). Similar situations were also observed with *O. viverrini* [16], *F. hepatica* [13], [28] and *S. japonicum* [15]. Teguments of platyhelminths including parasitic trematodes have crucial importance for the nutrient uptakes and immune evasion. The secretory granules scattered within the tegument are actively involved in the secretory trail [51]. The trematode proteins might be secreted through the signal peptide-independent secretion mechanism [52] or other unidentified pathway, which is intimately related to the secretory granules. Alternatively, some kinds of proteins, if not all, would be diffused/leaked into the incubation medium through damaged tegument membrane. Quality *versus* quantity should be carefully taken into consideration when evaluating parasites' ESP.

Recent advances in the analyses of the secretory proteome of helminth parasites combined with bioinformatics has immensely widened the understanding of human pathogens by allowing us to identify prodigious numbers of protein molecules, which are decisively involved in the pathobiological alterations [13]–[15], [28]. Our present study has demonstrated new aspects of host-parasite interactions. The secretory proteome of *C. sinensis* displayed common and unique features, which might be related to its habitat in the definitive host. The universal secretory proteins found in other parasites such as several enzymes involved in glucose metabolism and oxygen transporters were commonly identified in *C. sinensis* ESP, whereas cysteine proteases were not abundantly detected. Instead, *C. sinensis* secreted diverse GSTs, which might be importantly involved in the immunoprotection and detoxification of hydrophobic substances within bile ducts. Our data suggest strongly that different GSTs might have differentially evolved with the specialized functions. Cs28σGST1 and its much closer paralog(s) might play major roles as phase II antioxidant enzymes in intracellular compartments, while Cs28σGST3 and Cs26μGST2 may protect worms from exogenously derived toxic substances in extracellular phase, thus shaping the first-line defensive system.

## Supporting Information

Figure S1 **Protein profiles of** ***C. sinensis*** **ESP incubated with different dosages of host bile.** Adult worms (20 worms/group) were incubated in 3 ml RPMI-1640 alone or RPMI-1640 supplemented with rabbit bile (1, 5, 10 or 20%) or hydrogen peroxide (0.5 mM) at 37°C for 1 h. The incubation media were harvested and centrifuged at 20000 g for 30 min. The resulting supernatants were used as respective ESP. (**A**) The protein profiles contained in an equal volume (30 µl) of the ESP were examined by 12% SDS-PAGE under reducing conditions with CBB staining. (**B**) The relative intensities of visible protein bands were analyzed using a densitometer. An incubation of dead but uncorrupted worms in RPMI supplemented with 10% bile was included in the analysis as a control (lane Control).
(TIF)

Figure S2 **Primary and tertiary structures of Cs28σGST3 and its homologs.** (**A**) The amino acid sequences of σ-like GST proteins isolated from trematode species were aligned for the comparison of primary structure. Sigma-like GST proteins of *Necator americanus* (NaGST-2; 2ON5_A) and *Drosophila melanogaster* (DmGST-2; NP_725653), of which crystal structures have been empirically determined [20], [53], were also included in the analysis. Amino acid residues conserved were highlighted in black box and those in the glutathione- and substrate-binding pockets (G- and H-site) were marked by red and blue letters and circles, respectively. Arginine (R) residue involved in the stabilization of phenoxy ring of catalytic tyrosine (Y) residue is indicated by a green letter with circle. Secondary structure of Cs28GST3 predicted by the PredictProtein program in ExPASy and verified by simulation of tertiary structure is presented at the bottom of the alignment. Blue box indicates an amino acid stretch specifically observed in trematode proteins. (**B**) The tertiary structure of Cs28σGST3 (black) was predicted with the ESyPred3D program and aligned with those of the *N. americanus* (NaGST-2; dark gray) and *D. melanogaster* proteins (DmGST-2; light gray). The amino acids composing the G-site were marked by orange color and the conserved arginine was shown in green. Blue box demonstrates portion by amino acid stretch, which was found to be specifically conserved among members of trematode species. (**C**) Geometry of H-site was magnified to mark the amino acids associated with the binding of substrates in Cs28σGST3 and DmGST-2 proteins (red and blue colors, respectively).
(TIF)

Figure S3 **Biochemical properties of recombinant CsGST proteins.** The nucleotides corresponding to the full ORF of *Cs28σGST1*, *Cs28σGST3* and *Cs26μGST2* were cloned into pET-28a plasmid vector and the recombinant proteins were expressed in *E. coli* cells. The proteins were purified using Ni-NTA column and were subjected to the enzyme assay. The relative enzymatic activities according to pH of reaction buffer (**A**) and heat stability during time periods as indicated (**B**) as indicated were assayed using reduced glutathione (GSH) and 1-chloro-2,4-dinitrobenzene (CDNB). The inhibitory modulation of GST-specific inhibitors were compared in pH 7, and IC_50_ values of selected compounds were determined (**C**). Assays were performed in triplicates and IC_50_ values were calculated by non-linear regression analysis based on the approximate equation. % inhibition = 100 [I]/IC_50_+[ΣI] where the free inhibitor concentration [I] was initially approximated by the total inhibitor concentrations [ΣI].
(TIF)

Figure S4 **Phylogenetic position of Cs28σGST3.** Information on the proteins annotatable as GST was retrieved from the protostomian databases of GenBank. The amino acid sequences were used in the construction of the neighbor-joining tree with MEGA program. The tree was unrooted and the statistical significance of each branching node was estimated by a bootstrapping analysis (1000 replicates) of the initial input alignment. The family name of the antioxidant proteins was marked and proteins from *C. sinensis*, of which sequence information was used in the generation of recombinant proteins, were indicated by arrows. The taxonomical positions of donor organisms were distinguished by colored letter; red, platyhelminths; blue, nematodes; green, insects; brown, molluscans; black, arachnids; purple, crustaceans.
(TIF)

Table S1 **Primers used in this study.**
(DOC)

Table S2 **Putative identification of proteome of adult** ***C. sinensis*** **ESP and 10% bile juice followed by MALDI-TOF MS and tandem MS analysis.**
(DOC)
