# Supplementary figures and images for: Differential Activation of Diverse Glutathione Transferases of Clonorchis sinensis in Response to the Host Bile and Oxidative Stressors
Source: PLoS Negl Trop Dis. 2013 May 16;7(5):e2211. doi: 10.1371/journal.pntd.0002211 (PMC3656158; doi:10.1371/journal.pntd.0002211)

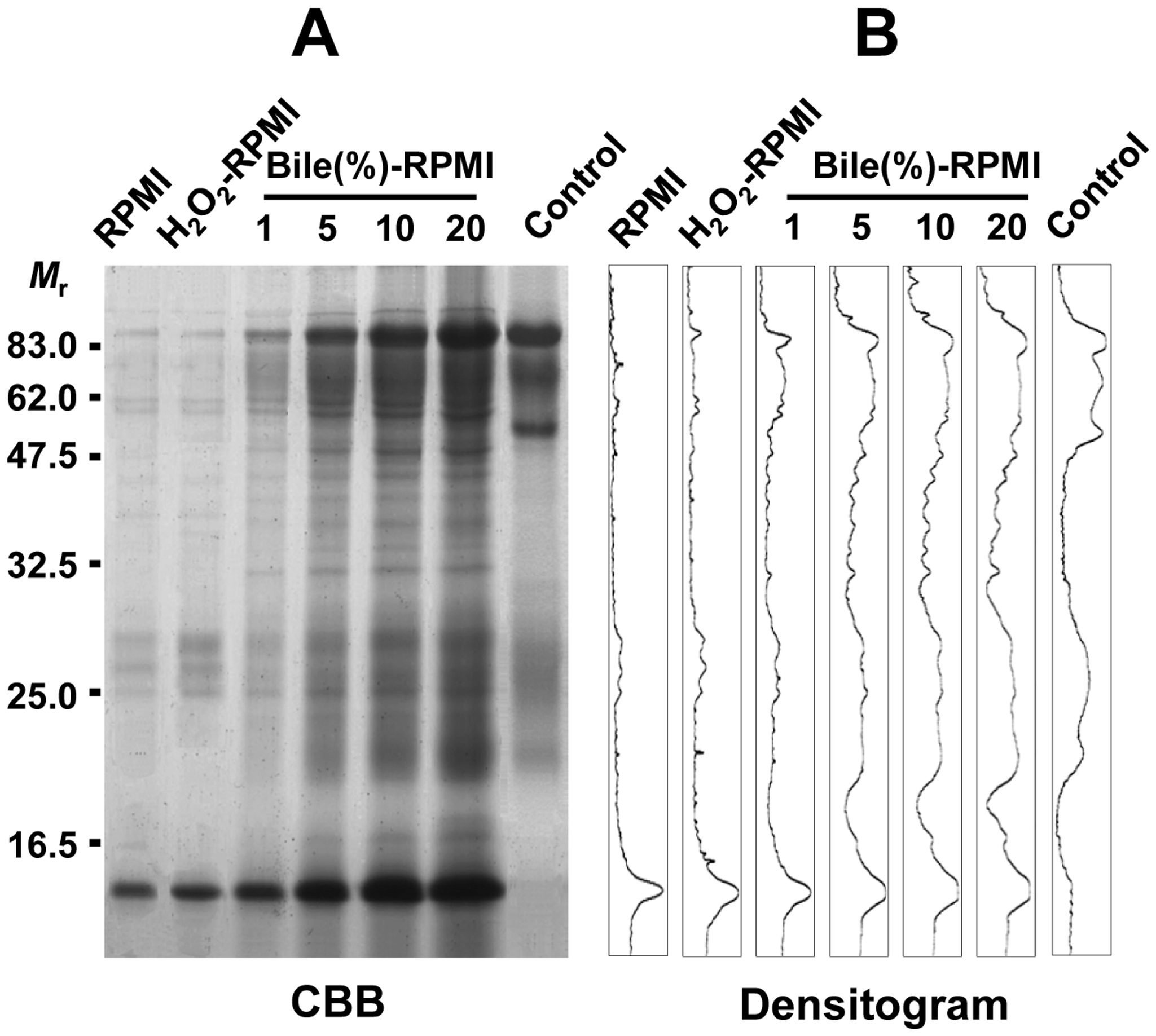

Supplement: Figure S1 — Protein profiles of C. sinensis ESP incubated with different dosages of host bile. Adult worms (20 worms/group) were incubated in 3 ml RPMI-1640 alone or RPMI-1640 supplemented with rabbit bile (1, 5, 10 or 20%) or hydrogen peroxide (0.5 mM) at 37°C for 1 h. The incubation media were harvested and centrifuged at 20000 g for 30 min. The resulting supernatants were used as respective ESP. (A) The protein profiles contained in an equal volume (30 µl) of the ESP were examined by 12% SDS-PAGE under reducing conditions with CBB staining. (B) The relative intensities of visible protein bands were analyzed using a densitometer. An incubation of dead but uncorrupted worms in RPMI supplemented with 10% bile was included in the analysis as a control (lane Control). (TIF) [file pntd.0002211.s001.tif]

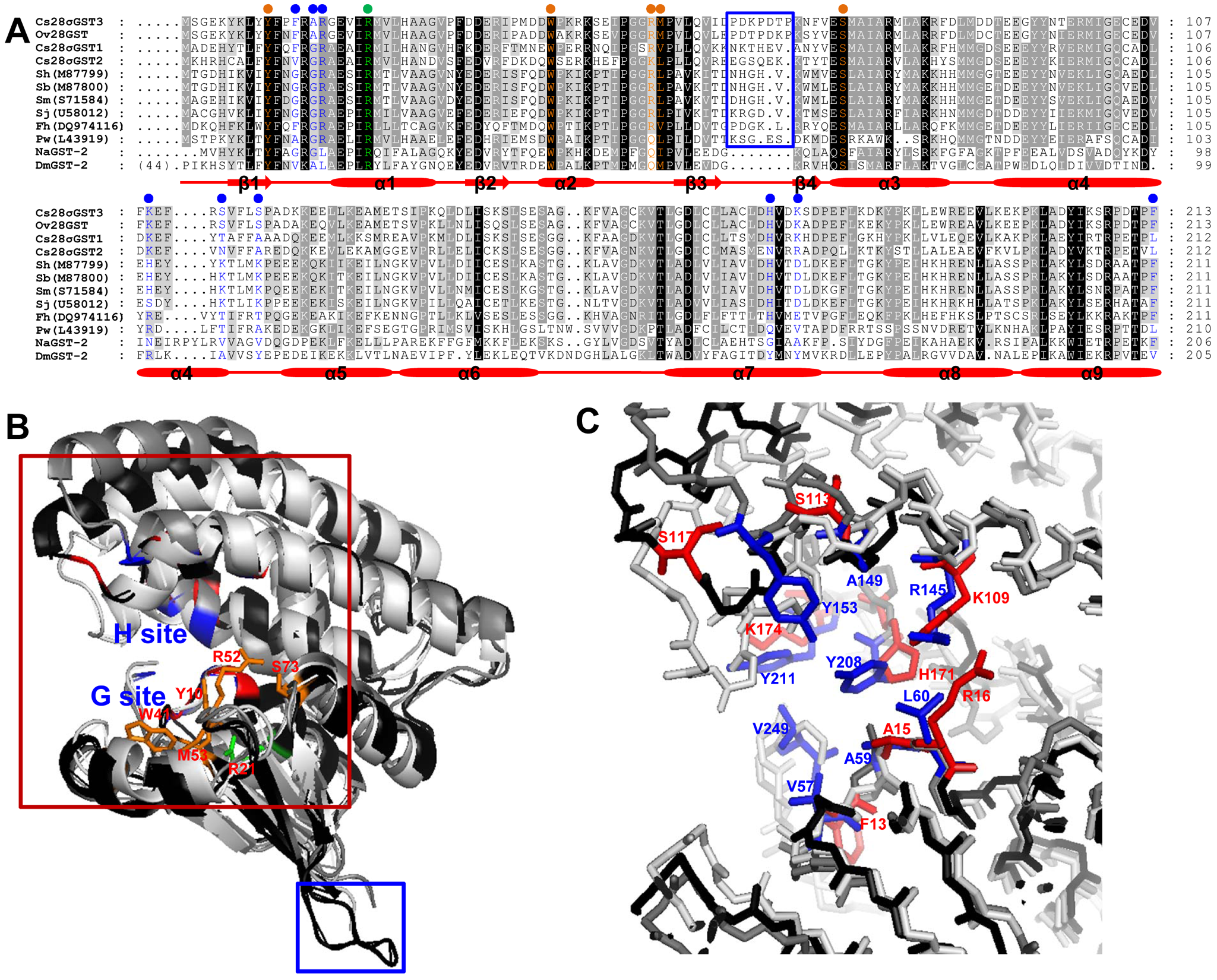

Supplement: Figure S2 — Primary and tertiary structures of Cs28σGST3 and its homologs. (A) The amino acid sequences of σ-like GST proteins isolated from trematode species were aligned for the comparison of primary structure. Sigma-like GST proteins of Necator americanus (NaGST-2; 2ON5_A) and Drosophila melanogaster (DmGST-2; NP_725653), of which crystal structures have been empirically determined [20], [53], were also included in the analysis. Amino acid residues conserved were highlighted in black box and those in the glutathione- and substrate-binding pockets (G- and H-site) were marked by red and blue letters and circles, respectively. Arginine (R) residue involved in the stabilization of phenoxy ring of catalytic tyrosine (Y) residue is indicated by a green letter with circle. Secondary structure of Cs28GST3 predicted by the PredictProtein program in ExPASy and verified by simulation of tertiary structure is presented at the bottom of the alignment. Blue box indicates an amino acid stretch specifically observed in trematode proteins. (B) The tertiary structure of Cs28σGST3 (black) was predicted with the ESyPred3D program and aligned with those of the N. americanus (NaGST-2; dark gray) and D. melanogaster proteins (DmGST-2; light gray). The amino acids composing the G-site were marked by orange color and the conserved arginine was shown in green. Blue box demonstrates portion by amino acid stretch, which was found to be specifically conserved among members of trematode species. (C) Geometry of H-site was magnified to mark the amino acids associated with the binding of substrates in Cs28σGST3 and DmGST-2 proteins (red and blue colors, respectively). (TIF) [file pntd.0002211.s002.tif]

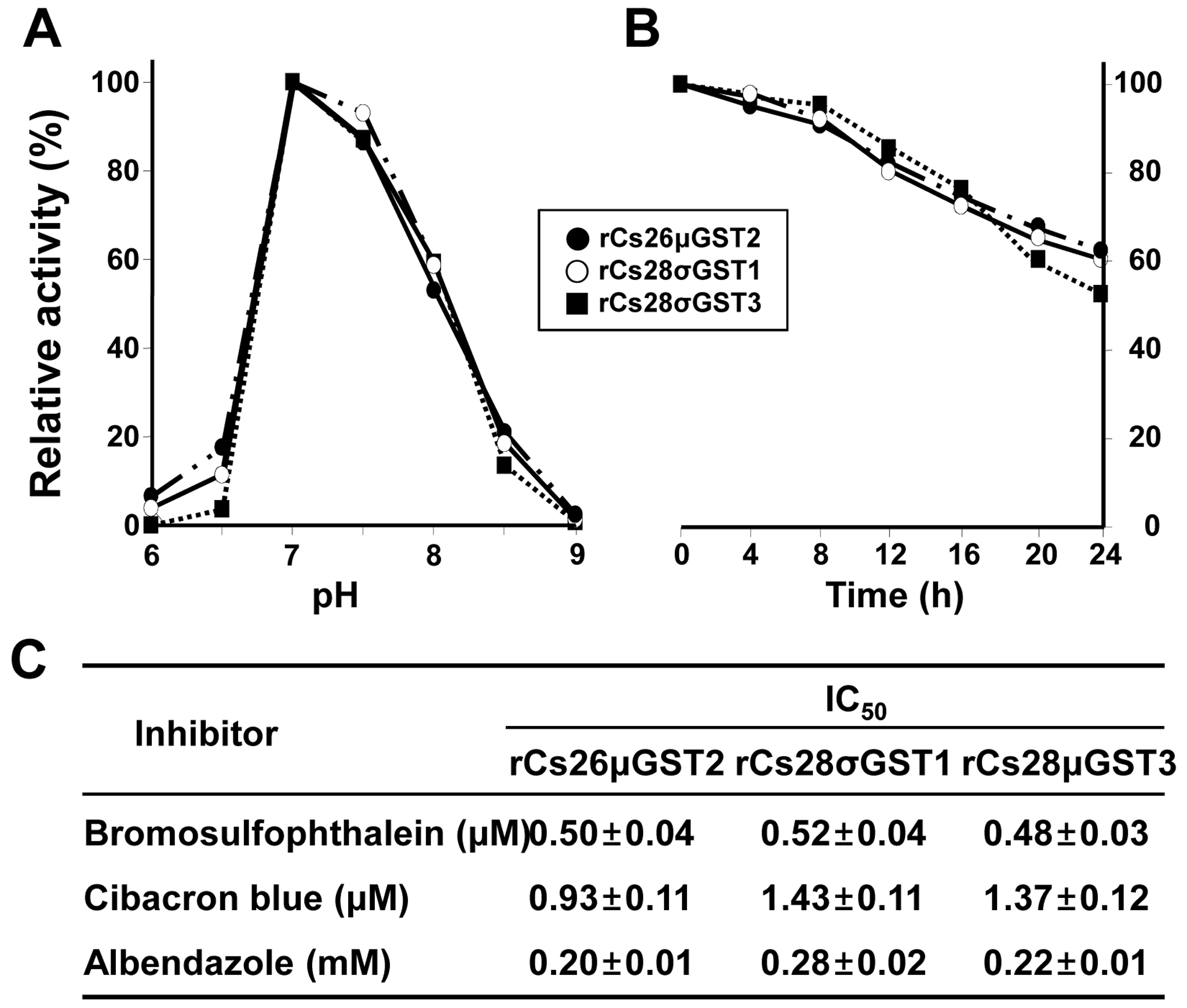

Supplement: Figure S3 — Biochemical properties of recombinant CsGST proteins. The nucleotides corresponding to the full ORF of Cs28σGST1, Cs28σGST3 and Cs26μGST2 were cloned into pET-28a plasmid vector and the recombinant proteins were expressed in E. coli cells. The proteins were purified using Ni-NTA column and were subjected to the enzyme assay. The relative enzymatic activities according to pH of reaction buffer (A) and heat stability during time periods as indicated (B) as indicated were assayed using reduced glutathione (GSH) and 1-chloro-2,4-dinitrobenzene (CDNB). The inhibitory modulation of GST-specific inhibitors were compared in pH 7, and IC50 values of selected compounds were determined (C). Assays were performed in triplicates and IC50 values were calculated by non-linear regression analysis based on the approximate equation. % inhibition = 100 [I]/IC50+[ΣI] where the free inhibitor concentration [I] was initially approximated by the total inhibitor concentrations [ΣI]. (TIF) [file pntd.0002211.s003.tif]

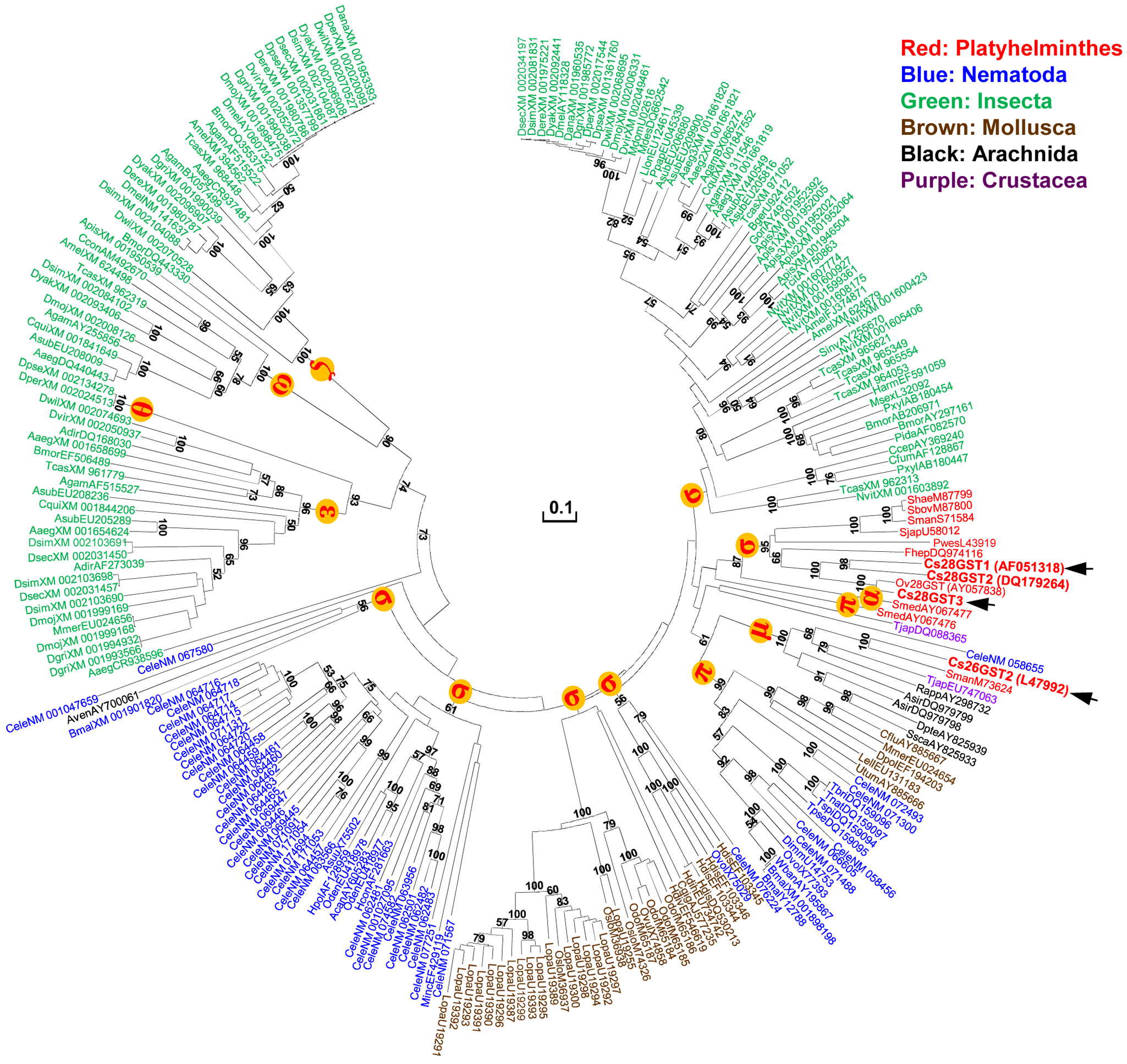

Supplement: Figure S4 — Phylogenetic position of Cs28σGST3. Information on the proteins annotatable as GST was retrieved from the protostomian databases of GenBank. The amino acid sequences were used in the construction of the neighbor-joining tree with MEGA program. The tree was unrooted and the statistical significance of each branching node was estimated by a bootstrapping analysis (1000 replicates) of the initial input alignment. The family name of the antioxidant proteins was marked and proteins from C. sinensis, of which sequence information was used in the generation of recombinant proteins, were indicated by arrows. The taxonomical positions of donor organisms were distinguished by colored letter; red, platyhelminths; blue, nematodes; green, insects; brown, molluscans; black, arachnids; purple, crustaceans. (TIF) [file pntd.0002211.s004.tif]
