# Supplementary material for: Differential Activation of Diverse Glutathione Transferases of Clonorchis sinensis in Response to the Host Bile and Oxidative Stressors
Source: PLoS Negl Trop Dis. 2013 May 16;7(5):e2211. doi: 10.1371/journal.pntd.0002211 (PMC3656158; doi:10.1371/journal.pntd.0002211)
Supplement: Table S1 — Primers used in this study. (DOC) [file pntd.0002211.s005.doc]

**Table S1.** Primers used in this study.

| Experiment | Name | Sequence (5'→3') |
| --- | --- | --- |
| Generation of recombinant  proteins | Cs26μGST2-Sal-F^a^  Cs26μGST2-Not-R^b^  Cs28σGST1-Sal-F  Cs28σGST1-Not-R  Cs28σGST3-Sal-F  Cs28σGST3-Not-R | AAAGTCGACAGCTGGAGTACGTCGGTGACAG  TAAGCGGCCGCTTATTTCGGAGGAGCATCGC  GGGGTCGACAGAGGATGATATTGCATGCAAA  TAAGCGGCCGCTTACAGGGGCGTTTCTGGTC  AAAGTCGACAGCGCATGGTGCTGCATGCAGC  TAAGCGGCCGCTCAGAAAGGTGTGTCAGGAC |
| Amplification  of chromosomal  gene | Cs28σGST1-g^c^-F  Cs28σGST1-g-R  Cs28σGST3-g-F  Cs28σGST3-g-R  Ov28GST-g-F  Ov28GST-g-R | ATATGGCTGACGAACACTACACGCTATTTTACTTTCA  CTCGTTACAGGGGCGTTTCTGGTCGTGTTCTG  CACCATGAGTGGCGAAAAATACAAGCTATACTATTTC  CAATCAGAAAGGTGTGTCAGGACGTGACTTTATGTA  ACCATGAGTGGTGAAAAATACAAGCTATACTATTTC  TCAGAAAGGTGTGTCAGGACGTGACTTTATGTAATC |
| *Cs28GST3*  genomic probes (Southern blotting) | Pr1^d^-F  Pr1-R  Pr2-F  Pr2-R  Pr3-Fs  Pr3-R  Pr4-F  Pr4-R  Pr5-F  Pr5-R  Pr6-F  Pr6-R | GTTGTTTTCACCATGAGTGGC  CACACTCTCACCTTTTCAGGTG  GATAGCTTTGATACCCGAGAGC  CTTGCAAGAGCCAATCGACGTTG  TGATGTTGTTAGCCAGATACTGG  CAGCTAGGAGTGACTGAACAGC  CAAGCCTTCCACCACCTGACTG  GCCAGCATATGCACAAAGGATG  TTACTAGTGACGACTGAACTCC  TCTAGAATGACCTGCAGAACTC  GTGAGTATACACAAGCCCATGG  GAACACACATGTGTAGACGTAC |
| Real-time PCR | CsAct^e^-os^f^-F  CsAct-os-R  Cs28σGST3-os-F  Cs28σGST3-os-R  Cs28σGST1-os-F  Cs28σGST1-os-R  Cs26μGST-os-F  Cs26μGST-os-R  CsSOD^g^-os-F  CsSOD-os-R | TGTCTTTCCGTCCATCGTTG  GCAGTTCATTGTAGAATGTATG  CACCGAAGAACTTTGTGGAG  GCTTCTTTCAAGAGTTCTTCC  TGATATTGCATGCAAACGGTG  GAATCACCCATCATATGGAAG  GTTTGTTGCTGGAGTACGTC  AGCAATGTAACGTAGAATGGC  GGATCCGGTTCGTCATGTC  AGTGGTTTGCTGAATTCGTG |

^a^F, forward.

^b^R, reverse.

^c^g, genomic.

^d^Pr, probe no.

^e^CsAct, *C. sinensis* actin.

^f^os, oxidative stress.

^g^CsSOD, *C. sinensis* superoxide dismutase.
