# Supplementary material for: Differential Activation of Diverse Glutathione Transferases of Clonorchis sinensis in Response to the Host Bile and Oxidative Stressors
Source: PLoS Negl Trop Dis. 2013 May 16;7(5):e2211. doi: 10.1371/journal.pntd.0002211 (PMC3656158; doi:10.1371/journal.pntd.0002211)
Supplement: Table S2 — Putative identification of proteome of adult C. sinensis ESP and 10% bile juice followed by MALDI-TOF MS and tandem MS analysis. (DOC) [file pntd.0002211.s006.doc]

**Table S2. Putative identification of proteome of adult *C. sinensis* ESP and 10% bile juice followed by MALDI-TOF MS and tandem MS analysis.**

| **Spot**  **no.** | **MS^a^** | **Mascot**  **score** | **Mat. pept.**  **(Total)^b^** | **Seq.**  **cov.^c^** | **Theoretical**  ***M*_r_/pI** | **NCBI no.^d^** | **Species** | **Description**  **(Matched sequences)** | **Sig.**  **pept.^f^** |
| --- | --- | --- | --- | --- | --- | --- | --- | --- | --- |
| **Proteins identified in 10%-bile ESP** | | | | | | | | | |
| 1 | TT | 97 | 3 | 6 | 73058/5.13 | AAK14176 | *O. felineus* | Paramyosin (m/z1973.83=324DLQNEVDSLSAENAELAR341  m/z1477.67=548VKDLEAFLEEER559  m/z1302.61=577 IQLSSEVEELR 587) | - |
| 2 | TT | 35 | 2 | 4 | 73058/5.13 | AAK14176 | *O. felineus* | Paramyosin  (m/z1973.84=324DLQNEVDSLSAENAELAR341  m/z1477.67=548VKDLEAFLEEER559) | - |
| 3 | MT | 80 | 11/31 | 43 | 20670/6.93 | EAT38124 | *A. aegyti* | Aldehyde dehydrogenase | - |
| 4 | MT | 80 | 11/31 | 43 | 20670/6.93 | EAT38124 | *A. aegyti* | Aldehyde dehydrogenase | - |
| 5 | TT | 118 | 2 | 3 | 83835/4.92 | P01832 | Rabbit | Polymeric immunoglobulin receptor (m/z1358.54=70DGHEFEEVAAVR86  m/z 1879.54=542CVTLASTGYTSQEYSGR553) | + |
| 6 | TT | 38 | 2 | 6 | 46245/6.28 | AAA57450 | *F. hepatica* | Enolase  (m/z1764.80=33AAVPSGASTGVHEALELR50  m/z1834.80=373SGETEDNFIADLVVGLR389) | - |
| 8 | TT | 147 | 2 | 8 | 46245/6.28 | AAA57450 | *F. hepatica* | Enolase  (m/z1764.80=33AAVPSGASTGVHEALELR50  m/z1834.80=373SGETEDNFIADLVVGLR389) | - |
| 9 | TT | 261 | 3 | 9 | 46245/6.28 | AAA57450 | *F. hepatica* | Enolase (m/z1764.80=33AAVPSGASTGVHEALELR50  m/z1834.80=373SGETEDNFIADLVVGLR389  m/z806.40=407YNQLLR412) | - |
| 10 | TT | 54 | 2 | 8 | 46245/6.28 | AAA57450 | *F. hepatica* | Enolase (m/z1764.80=33AAVPSGASTGVHEALELR50  m/z1834.80=373SGETEDNFIADLVVGLR389412) | - |
| 11 | MT | 82 | 8/21 | 15 | 75090/5.22 | XP_001293860 | *T. vaginalis* | Hypothetical protein (TVAG_143150) | - |
| 12 | TT | 257 | 5 | 8 | 83835/4.92 | P01832 | Rabbit | Polymeric immunoglobulin receptor  (m/z1931.82=278GGNVVIDSQGTIDPAFEGR296  m/z1469.69=302AENGHFSVVIAGLR315  m/z990.47=359GFPGGSVTIR368  m/z1394.67=392HLLVDSGEGLVQK404  m/z1358.54=542DGHEFEEVAAVR553) | + |
| 14 | TT | 65 | 6 | 21 | 44118/7.74 | ABU86400 | *C. sinensis* | Myosin heavy chain  (m/z1179.61=88K.AGTLASLEDMR.D98  m/z1557.85=193K.ELEEQNVTVLQQK.N205  m/z943.60=226K.ISNLVLQR.G233  m/z1701.90=298R.TLQGEIAQQDEQITK.L312  m/z1966.99=327RTQEALQAEEDKVNHLNKL343  m/z1864.92=346K.AKLESTLDEMEENLAR.E361) | - |
| 15 | TT | 34 | 2 | 8 | 83835/4.92 | P01832 | Rabbit | Polymeric immunoglobulin receptor  (m/z1393.67=392HLLVDSGEGLVQK404  m/z1358.53=542DGHEFEEVAAVR553) | + |
| 16 | TT | 37 | 2 | 8 | 36520/5.20 | AAF40479 | *C. sinensis* | Cathepsin  (m/z1891.75=42TYSNDDDELRFEIFK56  m/z1692.58=293NSWGEDFGEEGYFR306) | + |
| 17 | MT | 36 | 3/8 | 23 | 36355/8.16 | AAW25322 | *S. japonicum* | Glyceraldehyde 3-phosphate dehydrogenase | - |
| 18 | MT | 81 | 7/21 | 23 | 51537/5.40 | NP_274496 | *N. meningitidis* | Succinate-semialdehyde dehydrogenase | - |
| 19 | MT | 92 | 7/15 | 25 | 36432/8.57 | AAT46071 | *C. sinensis* | Cytosolic malate dehydrogenase | - |
| 21 | MT | 82 | 6/14 | 38 | 21813/5.76 | CAG05601 | *T. nigroviridis* | LOH1CR12^g^ | - |
| 22 | MT | 94 | 11/33 | 46 | 24735/5.22 | ABC72085 | *C. sinensis* | Glutathione transferase (σ) **Cs28σGST3**^h^ | - |
| 23 | MT | 77 | 7/25 | 23 | 25470/6.74 | CAA59739 | *E. multilocularis* | Glutathione transferase (μ1) **Cs26μGST1** | - |
| 24 | MT | 89 | 7/17 | 28 | 34814/6.49 | AAO49385 | *S. mansoni* | Glutathione transferase (ω) **Cs28ωGST** | - |
| 25 | MT | 98 | 7/13 | 32 | 34814/6.49 | AAO49385 | *S. mansoni* | Glutathione transferase (ω) **Cs28ωGST** | - |
| 26 | MT | 50 | 5/14 | 13 | 48399/4.78 | AAX24457 | *S. japonicum* | SJCHGC05999 | - |
| 27 | MT | 102 | 7/15 | 40 | 25756/4.97 | BAA84280 | *C. sinensis* | Cathepsin F | + |
| 28 | MT | 50 | 5/14 | 13 | 27064/8.18 | XP_002571861 | *S. mansoni* | Triosephosphate isomerae | - |
| 29 | MT | 93 | 8/24 | 41 | 27064/8.18 | XP_002571861 | *S. mansoni* | Triosephosphate isomerae | - |
| 30 | MT | 97 | 7/17 | 27 | 37019/5.29 | AAP33049 | *C. sinensis* | Cathepsin L | + |
| 31 | MT | 44 | 3/7 | 19 | 25756/4.97 | BAA84280 | *C. sinensis* | Cathepsin F | + |
| 32 | MT | 104 | 10/24 | 42 | 24559/6.97 | ABA56496 | *C. sinensis* | Glutathione transferase (σ) **Cs28σGST2** | - |
| 33 | MT | 121 | 10/19 | 35 | 24775/8.40 | AAD17488 | *C. sinensis* | Glutathione transferase (σ) **Cs28σGST1** | - |
| 34 | MT | 170 | 15/31 | 56 | 24775/8.40 | AAD17488 | *C. sinensis* | Glutathione transferase (σ) **Cs28σGST1** | - |
| 35 | MT | 76 | 5/9 | 31 | 25080/6.07 | AAB46369 | *C. sinensis* | Glutathione transferase (μ2) **Cs26μGST2** | - |
| 36 | TT | 97 | 6 | 36 | 25080/6.07 | AAB46369 | *C. sinensis* | Glutathione transferase (μ2) **Cs26μGST2**  (m/z2029.77=19RLLLEYVGDSYEEHSYGRC35  m/z1670.73=46 KHNLGLELPNLPYYKD59  m/z1534.68=60KDGNFSLTQSLAILR.Y73  m/z1318.57=92KISMIEGGLVDLR.A103  m/z1369.57=109RIAYQETFEQLKV119  m/z1414.67=120KVPYLQQLPSTLRM131) | - |
| 37 | MT | 119 | 9/22 | 50 | 25080/6.07 | AAB46369 | *C. sinensis* | Glutathione transferase (μ2) **Cs26μGST2** | - |
| 38 | MT | 114 | 11/32 | 52 | 19596/5.51 | AAS92978 | *C. sinensis* | Ferritin | - |
| 39 | TT | 55 | 3 | 36 | 10949/9.85 | AAX30173 | *S. mansoni* | SJCHGC01414  (m/z1494.76=1VSALLVSMPKYDRQ13  m/z1229.82=20RVFVGGVDPRVGKV31  m/z1179.63=84RLRVEHAVNNKT93) | - |
| 40 | MT | 64 | 6/23 | 42 | 16985/5.14 | AAM18464 | *C. sinensis* | Myoglobin | - |
| 41 | MT | 135 | 12/32 | 57 | 20490/9.45 | AT008124 | *C. sinensis* | Fatty acid binding protein | - |
| **Proteins identified in 10% bile juice** | | | | | | | | | |
| a | TT | 171 | 3 | 5 | 84975/4.92 | P01832 | Rabbit | Polymeric immunoglobulin receptor  (m/z1879.85=70RCVTLASTGYTSQEYSGRG86  m/z990.54=359KGFPGGSVTIRC368  m/z1358.63=542KDGHEFEEVAAVRV553) | + |
| b | MT | 262 | 20/27 | 42 | 70861/5.85 | NP_001075813 | Rabbit | Serum albumin precursor | + |
| c | MT | 84 | 11/36 | 20 | 84975/4.92 | P01832 | Rabbit | Polymeric immunoglobulin receptor | + |
| d | MT | 107 | 8/14 | 27 | 36213/5.42 | NP_077069 | Rat | Annexin A4 | - |
| e | MT | 81 | 6/23 | 41 | 24097/7.64 | EDM10622 | Rat | Junction adhesion molecule 2 | + |
| f | MT | 43 | 4/19 | 30 | 16003/4.72 | P23108 | Rabbit | Immunoglobulin J chain | - |

^a^Mass spectra were observed by MALDI-TOF/TOF (TT) or MALDI-TOF (MT).

^b^Values are shown by matched peptides/total peptides.

^c^Sequence coverage (%)

^d^GenBank accession numbers (Protein number)

^e^Protein functions based on Gene Ontology of UniProtKB ([http://uniprot.org](http://uniprot.org/)).

^f^Presence of signal peptide sequence predicted by the SignalP 4.0 (<http://www.cbs.dtu.dk/services/SignalP/>) and PSORT (http://psort.nibb.ac.jp).

^g^Loss of heterozygosity, 12, chromosomal region 1 protein homolog

^h^*C. sinensis* GSTs renamed in this study by considering their identification order, biochemical properties, phylogenetic positions and locations on 2-DE gel are indicated by bold characters.
